# Novel approach to analysis of the immune system using an ungated model of immune surface marker abundance to predict health outcomes

**DOI:** 10.1186/s12979-022-00291-y

**Published:** 2022-08-04

**Authors:** G. Provost, F. B. Lavoie, A. Larbi, TP. Ng, C. Tan Tze Ying, M. Chua, T. Fulop, A. A. Cohen

**Affiliations:** 1 Groupe de recherche PRIMUS, Department of Family Medicine, University of Sherbrooke, 3001 12e Ave N, QC J1H 5N4 Sherbrooke, Canada; 2 Singapore Immunology Network (SIgN), Agency for Science Technology and Research (A*STAR), Immunos Building, Biopolis, Singapore, Singapore; 3 Gerontology Research Programme, Department of Psychological Medicine, Yong Loo Lin School of Medicine, National University Health System, National University of Singapore, Singapore, Singapore; 4 Research Center on Aging, Geriatric Division, Department of Medicine, Faculty of Medicine and Health Sciences, Université de Sherbrooke, QC Sherbrooke, Canada

**Keywords:** Immunology, Neural network, Complex system

## Abstract

Traditionally, the immune system is understood to be divided into discrete cell types that are identified via surface markers. While some cell type distinctions are no doubt discrete, others may in fact vary on a continum, and even within discrete types, differences in surface marker abundance could have functional implications. Here we propose a new way of looking at immune data, which is by looking directly at the values of the surface markers without dividing the cells into different subtypes. To assess the merit of this approach, we compared it with manual gating using cytometry data from the Singapore Longitudinal Aging Study (SLAS) database. We used two different neural networks (one for each method) to predict the presence of several health conditions. We found that the model built using raw surface marker abundance outperformed the manual gating one and we were able to identify some markers that contributed more to the predictions. This study is intended as a brief proof-of-concept and was not designed to predict health outcomes in an applied setting; nonetheless, it demonstrates that alternative methods to understand the structure of immune variation hold substantial progress.

**Supplementary Information:**

The online version contains supplementary material available at 10.1186/s12979-022-00291-y.

## Introduction

In recent decades, aspects of immune function have been linked to the outcomes of an increasing number of medical conditions, including cancer [1], diabetes [2], Alzheimer’s disease [3], and cardiovascular diseases [4]. This indicates that the immune system is at the forefront of our fight against not only infectious diseases but also a wide range of other conditions: the immune system communicates with, and is an integral part of, global physiological networks that maintain dynamic equilibrium [5, 6], and immune perturbations, dysregulations, and adaptations can have wide-ranging effects [7–9]. Accordingly, changes in immune state are a crucial feature of the aging process, and likely can contribute to links between aging and age-related disease. In this context, understanding how immune state interacts with other systems is crucial to a broad understanding of how organisms maintain dynamic equilibrium, and of how changes in immune state might contribute to or mitigate aging processes. Precise and valid measures of age-associated changes in immune state are thus a prerequisite both to a sufficient understanding of immunosenescence and to potential clinical applications, such as identifying appropriate immunotherapy regimes for patients [10]. In turn, such precise and valid measures of immune aging require an appropriate way to characterize the immune system more generally.

The current paradigm in immunology is to consider the immune system as composed of a multitude of specialized cells which can be classified into discrete types, each having precise roles in the defense of the organism [11–13]. The main way to analyze those cell types and their interactions is by flow cytometry because it allows for the classification by type and quantification of a large number of cells within an organism. The classical way to process the data produced by this technique into comprehensive information is called gating, which is a technique where the cells are placed on two-by-two grids of pairs of surface markers in a sequential manner to classify them [14]. However, this technique is imperfect: it results in the loss of some multivariate relationships among markers, in the loss of information on levels of cell surface markers, and in the exclusion or misplacement of some cellular populations due to the subjectivity of the technique and the rigid nature of its cut-off [15].

Other methods have been developed to overcome those shortcomings such as viSNE [16] or SPADE [17] which uses semi-supervised clustering methods to categorize cell types using multiple surface markers at the same time. This creates a more flexible way to separate the diverse cell types while conserving the multivariate structure of the data. However, these techniques also have disadvantages such as a lack of reproducibility between runs (SNE) or between algorithms and a lack of an unbiased way to decide if findings made by the algorithms are indeed findings or artefacts [18]. So, there is a need to develop other techniques to analyze immune cytometry data that could overcome these flaws.

In recent studies, it has been shown that immune cell subpopulations are more heterogeneous than was previously believed [19–21]. This heterogeneity is even further increased during the aging process, partially driven by naïve T cells, which are functionally different when generated at different stages of life [22, 23]. This suggests that immune cell types are less well-defined than was previously believed and has highlighted our lack of understanding of how many different immune cell subtypes might exist and what their precise roles and range of actions are. Despite these findings, there are hardly any studies analyzing the immune system without dividing it into cell types.

Here, we propose a different way of looking at the immune system, which is to directly analyze the values of the surface markers without dividing the cells into different subtypes. This might allow a more global view of the immune system and a less biased way to analyze it since no prior knowledge of the subdivision is assumed. Our goal is to assess if it is possible to obtain relevant biological information using only raw surface marker levels. An ideal method would include both continuous, multivariate information on cell surface markers and the identity of the cells they are on; this is, however, methodologically, and conceptually challenging, so our goal was to assess whether there was potential in going beyond traditional gating methods. The use of immune markers to get relevant biological information is not new [24, 25] and we expect that since the markers used to differentiate cell types have specific functions – beyond their ability to classify cells into discrete types – their overall levels could have an impact on health and immune system functioning without having to consider which cell they are on. To test this hypothesis, we used cytometry data from the Singapore Longitudinal Aging Study (SLAS) database and analyzed the distributions of 27 surface markers. We then performed nonlinear regression using two different neural networks to try to predict the presence of several health conditions and found that a model using raw surface marker abundance outperformed a model built using classically gated cell types. We also showed that there was no specific marker that contributed significantly more to the predictions. This study is intended as a brief proof-of-concept and was not designed to predict health outcomes in an applied setting.

## Methods

### Dataset

For all the analysis performed in this article, we used the second cohort of the Singapore Longitudinal Ageing Study (SLAS-2), a longitudinal study of aging and health of community-dwelling Singaporeans aged 55 or more at the start of the study, as previously described [26–28]. It excludes individuals unable to participate because of severe physical or mental disabilities. It includes 3200 residents of the southwest and central south of Singapore starting in 2010. The study received ethical approval from the National University of Singapore Institutional Review Board and written consent was obtained from all participants (response rate of 78%). The study followed the Strengthening the Reporting of Observational Studies in Epidemiology reporting guidelines [29]. Although the dataset presents longitudinal components, the flow cytometry data needed for this study were only available cross-sectionally in all participants. Data informations can be found in Table 1.

**Table 1:** Sample characteristics

	*n* = 567
**Age**	
Mean ± SD	67.1 ± 7.5
Range (min-max)	55–89
**Sex**	
M (%)	337 (39)
F (%)	527 (61)
**Mortality (%)**	33 (5.8)
**Self-assessed Health**	
1 (%) – better health	7 (1.2)
2 (%)	88 (15.5)
3 (%)	334 (58.9)
4 (%)	134 (23.6)
5 (%) – worst health	4 (0.7)
**Frailty**	
0 (%)	261 (46)
1 (%)	184 (32.5)
2 (%)	81 (14.3)
3 (%)	35 (6.2)
4 (%)	5 (0.9)
5 (%)	1 (0.2)
**MMSE, mean ± SD**	27.8 ± 2.8
**Comorbidity, mean ± SD**	2.4 ± 1.6
**High blood pressure (%)**	245 (43.2)
**High cholesterol (%)**	263 (46.4)
**Diabetes (%)**	77 (13.6)
**Stroke (%)**	23 (4)
**Heart attack (%)**	30 (5.3)
**Atrial fibrillation (%)**	19 (3.4)
**Eye problem (%)**	175 (30.1)
**Asthma (%)**	28 (4.9)
**Arthritis (%)**	80 (14.1)
**Osteoporosis (%)**	28 (4.9)
**Gastrointestinal problem (%)**	50 (8.8)
**Thyroid problem (%)**	28 (4.9)
**Cancer (%)**	19 (3.4)
**Depression (%)**	18 (3.2)

### Health outcome metrics

In our analysis, we looked at the predictive power of our models on 20 health or health-status-related measures: (1) Age; (2) Mortality; (3) Self-assessed health measured on a five-point Likert scale, based on the question “Generally would you say your health is: Excellent, Very good, Good, Fair or Poor”; (4) Frailty evaluated on the 5 criteria from Fried’s phenotypic scale [30]: weakness, slowness, weight loss, low physical activity and exhaustion [31]; (5) Global cognitive function as quantified via the Mini Mental State Evaluation (MMSE) [32]; (6) The number of comorbidities from a list of 23 based on self-report, medication and physical or laboratory tests; (7) High blood pressure; (8) High cholesterol; (9) Diabetes; (10) Stroke; (11) Heart attack; (12) Atrial fibrillation; (13) Eye problem; (14) Asthma; (15) Arthritis; (16) Osteoporosis, (17) Gastrointestinal problems; (18) Thyroid problems; (19) Cancer; (20) Depression. Most of these metrics are dichotomic, but age, self-assessed health, frailty, MMSE, and comorbidities are discrete measures with multiple values. Religion was also included as a negative control.

### Cell surface markers

The surface markers used in this article are 6-Sulfo LacNAc (Slan), CD19, Pan-GDT, TCRVg1, TCRVa7.2, CD45RO, CD127, CD56, HLADR, CCR6, CD45, CRTH2, CD34, CD38, CD57, CD25, CD16, CD123, CD27, CD3, CD8, CD14, CXCR3, TCRVg2, IgD, CD4 and CD161. The markers CD19 & Pan-GDT, TCRVg1 & TCRa7-2, CD8 & CD14, and TCRVg2 & IgD were paired together respectively on the same channel, during the panel design Flow Cytometry, as these markers are located on different cell types (mutually exclusive). Theses markers were selected because they allow for a good separation of a large number of immune cells subtypes, including but not limited to T cells, B cells, basophile, monocyte, NK cells and innate lymphoid cells. CD 57 was added as a marker of senescent cells.

### Preprocessing and statistical analysis

The Flow Cytometry data were analyzed with primary gating to exclude debris using the FSC-A/SSC-A gate, the FSC-A/FSC-H gate to keep only single cells and excluding cells absorbing the LIVE/DEAD™ Fixable Blue Stain (ThermoFisher Scientific). Finally, cells expressingCD45 + were kept. This gating enabled to work on single living leukocytes for the rest of the analyses.

For the non-gated model, since the number of cells varied between individuals but could often approach half a million, we randomly sampled 5000 cells for each individual to ensure equal representation and reduce computational time. Before this sampling, the first and last 10% of each individual file were removed to limit inconsistencies during the Flow Cytometry acquisition. Since a few extreme negative outliers were observed for most of the markers, a threshold was set at -50 000 relative fluorescence units for all markers and all cells with markers below that limit were removed. This was done to prevent these outliers from weighing too much on the model, since it is based on distribution. Then, for each individual, the distribution of fluorescence intensity of each marker was divided into 102 different sections. All values below the 2.5th percentile and above the 97.5th percentile were put together into the two lowest and highest sections, respectively, in order to avoid outliers having too strong of an impact on the results. The rest of the distributions were separated into 100 sections of the same width on the absolute scale. The number of cells present in each of these sections was then stored and used as input in the model. The individuals were split into groups of 300 for the calibration and 267 for the validation.

**Fig. 1:**
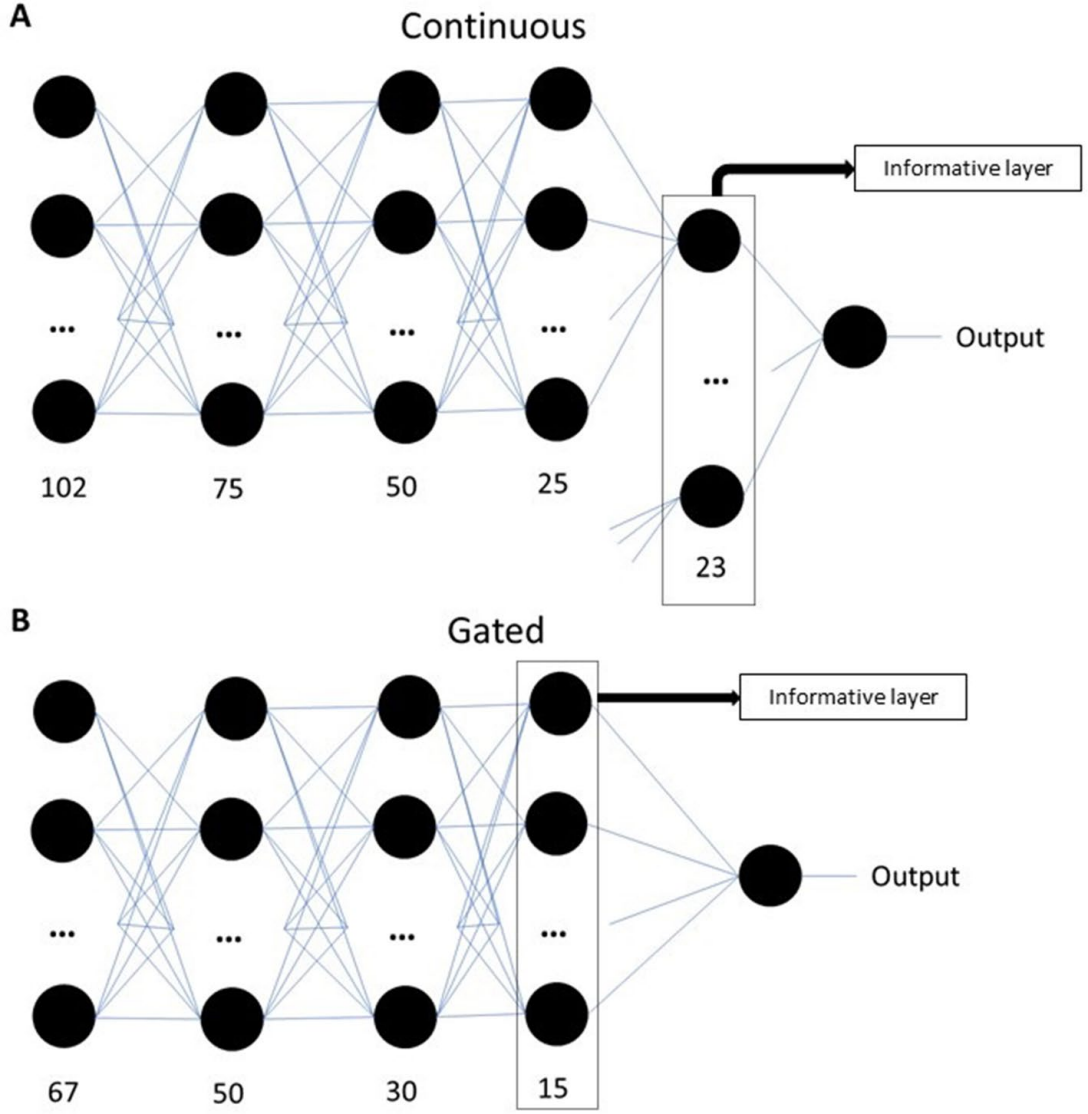
Representation of the models.** A** The continuous model. **B** The gated model

The non-gated model is therefore composed of 23 sets (one for each surface marker) of 102 inputs, each followed by a dense layer of 75 neurons, another dense layer of 50 neurons, another dense layer of 25 neurons, and then a dense layer of 1 neuron for the marker studied. The number of neurons in each layer was selected to be lower than the initial input layer and to form a decreasing gradient so that the later layers represent more generalized patterns. The last 23 layers of 1 neuron are then added and passed to a last dense layer of 1 neuron which gives the final output (Fig. 1A). For the first 3 layers of 75, 50, and 25 neurons, the activation function is the exponential linear unit and for the two layers of 1 neuron, the activation function is linear. The non-gated model was run with an epochs of 25,000 and a batch size of 100.

For the gated model, 67 mutually exclusive different cell types were obtained via a gating strategy shown in Additional file 2. The individuals were split into 300 for the calibration and 267 for the validation. The model is composed of 67 inputs, followed by a dense layer of 50 neurons, a dense layer of 30 neurons, a dense layer of 15 neurons, and a dense layer of 1 neuron which gives the final output (Fig. 1B). The first three layers of 50, 30, and 15 neurons have an exponential linear unit activation function and the last layer of 1 neuron has a linear activation function. The same reasoning as for the Continuous model was applied to the selection of the number of neurons in each layer for this model. For the gated model, an epochs of 10,000 was used since the model converged more easily and a batch size of 100.

For both models, individuals that had missing data in any of the measures were removed to keep the number of people used to calibrate and evaluate each model the same. Models were generated 100 different times using the same settings to create replicates to consider the random variation that can occur during the generation of the model. Both models used the Adam algorithm for there optimisation. Neural network models were chosen because it can model complex nonlinear relationship which is likely the case with immune data and the immune system in general. Additionally, neural network does not impose restriction on the input variables, which was useful to create our two models since both have very different input variables. All analyses were conducted using R v3.6.3 [33], Python 3.7.6 [34] and TensorFlow 1.14.0 [35].

Success of predictions was assessed based on the comparison of the root mean squared error (rmse) score and the mean value. An rmse for a health measure with no predictive capacity would be close to or higher than its mean value. Successful predictions were considered to be health measures for which the rmse was less than one third of the mean value.

## Results

### Distributions of the surface markers

**Fig. 2:**
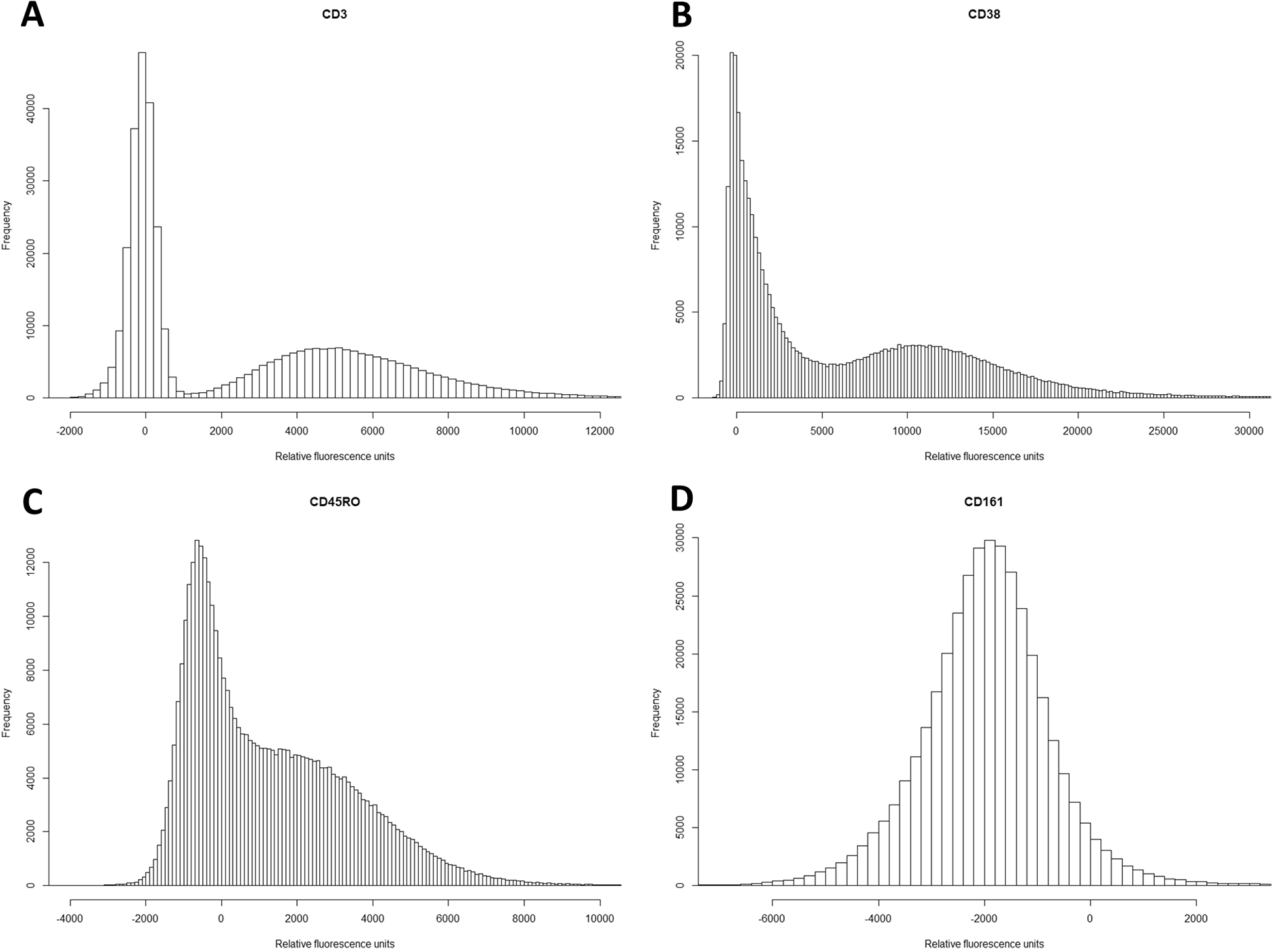
Example distributions of four of the surface markers tested in this article.** A** Distribution of CD3, used to identify T cells. **B** Distribution of CD38, used to identify B cell subsets. **C** Distribution of CD45RO, used to identify memory T cells. **D** Distribution of CD161, which can help define various T cell subsets

Figure 2 shows four representative examples of surface marker distributions. CD3 (Fig. 2A) is a clear bimodal distribution, presumably indicating a general presence (right) or absence (left) of the marker; note, however, the substantial quantitative variation in the marker on cells which are positive for it. CD 161 (Fig. 2D) is closer to a normal distribution indicating a more continuous gradient of abundance of the surface marker. This second type of distribution was more common amongst the markers analyzed, but some distributions also fell in between those categories like CD38 (Fig. 2B) and CD45RO (Fig. 2C). All distributions can be found in the supplement (Additional file 1).

### Testing the predictive capacity of the raw surface marker abundance

The predictive power of the model using raw surface marker abundance (continuous) and the one using gated cell types (gated) were determined using multiple health measures (Table 2). For most health measures, we were unable to obtain any successful prediction for either the gated or ungated models, which is not unexpected, as it would be surprising if immune markers were able to predict everything we tested. Nevertheless, we were able to successfully predict three health measures: Age, Self-assessed Health, and MMSE. All three scored low rmse for the validation set in comparison with their mean values, which would be the expected value for random noise with no predictive capacity for this health measure. Religion was added as a negative control to test for overfitting. Unfortunately, we don’t have any variables that can serve as an easy positive control.

**Table 2:** Averages and standard deviations of the rmse on the validation set of 100 separate runs of the non-gated and gated model for the health measure tested and the mean values for these measures. In bold are the health measures for which the models were able to make successful predictions (rmse < mean/3)

	Continuous		Gated		Mean
	**rmse**	**std rmse**	**rmse**	**std rmse**	
**Age**	**8.705**	**0.336**	**13.732**	**0.961**	**67.105**
Mortality	0.255	0.022	0.264	0.022	0.058
Religion	2.208	0.104	2.335	0.109	2.260
**Self-assessed Health**	**0.840**	**0.030**	**0.961**	**0.046**	**3.072**
Frailty	1.154	0.048	1.302	0.057	0.830
**MMSE**	**3.344**	**0.332**	**4.497**	**0.308**	**27.835**
Comorbidity	1.982	0.101	2.291	0.127	2.322
High blood Pressure	0.616	0.029	0.679	0.034	0.432
High cholesterol	0.626	0.024	0.714	0.037	0.464
Diabetes	0.413	0.026	0.481	0.034	0.136
Stroke	0.234	0.020	0.243	0.02	0.040
Heart attack	0.277	0.021	0.305	0.025	0.053
Atrial fibrillation	0.220	0.022	0.234	0.017	0.034
Eye problem	0.594	0.022	0.611	0.032	0.301
Asthma	0.263	0.020	0.298	0.032	0.049
Arthritis	0.428	0.022	0.481	0.023	0.141
Osteoporosis	0.277	0.035	0.301	0.027	0.049
Gastrointestinal problem	0.352	0.026	0.367	0.037	0.088
Thyroid problem	0.275	0.027	0.287	0.027	0.049
Cancer	0.219	0.025	0.216	0.017	0.034
Depression	0.214	0.020	0.241	0.047	0.032

**Fig. 3:**
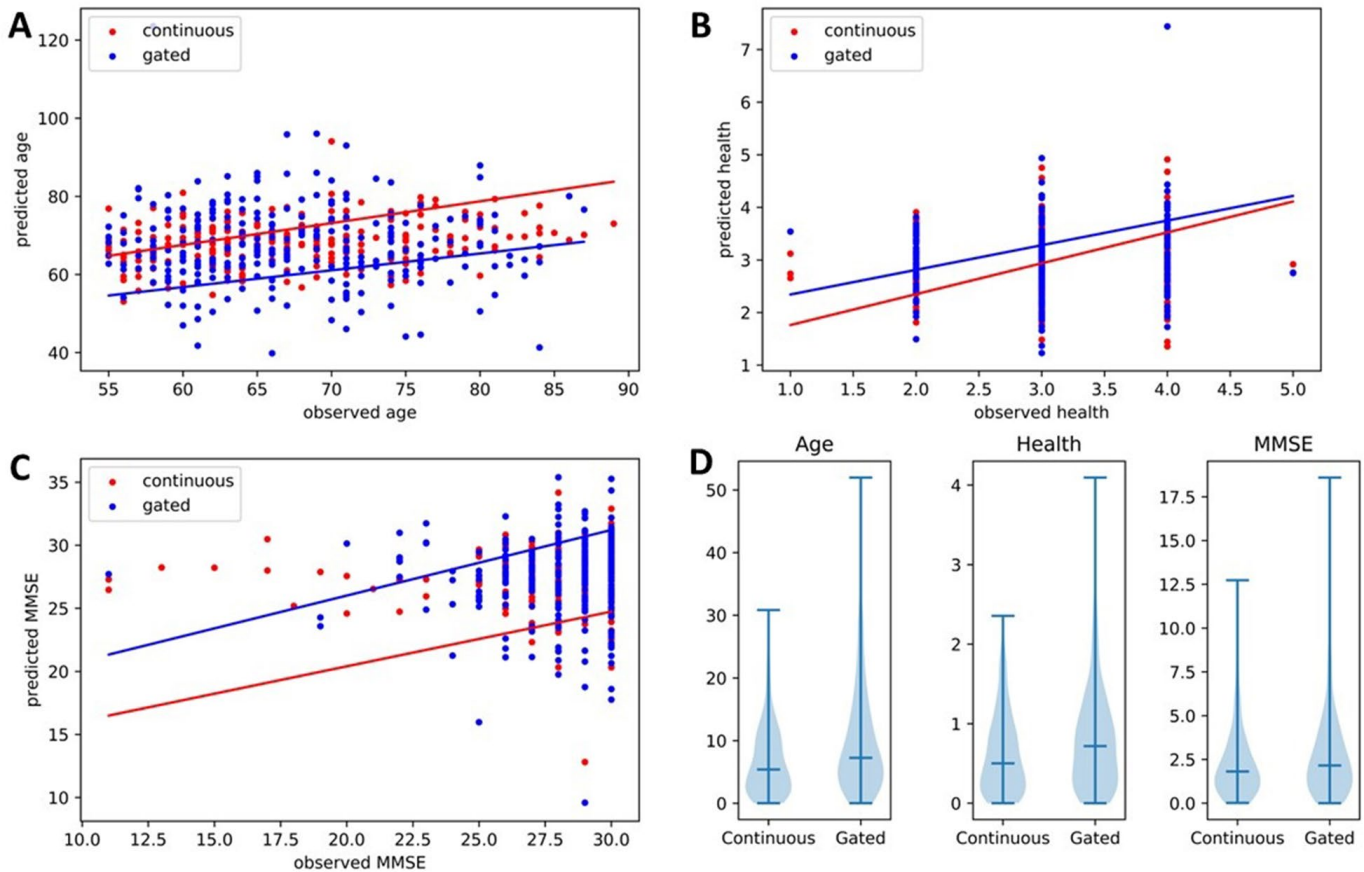
Comparison of the errors of the predictions between the continuous and the gated models for Age, Self-assessed health, and MMSE. **A**,** B** and** C** Scatter plots of the difference between the observed value and the predicted value for Age, Self-assessed health, and MMSE respectively. **D** Violin plot of the difference between the observed value and the predicted value, with the middle bar representing the median

The same health measures were significantly predicted in both models, but the rmse scores were higher in all three cases for the gated model, indicating a less precise prediction. It can be seen in the scatter plot of Fig. 3A, B, and C, as the errors of the gated model (in blue) seem to be more extreme. This is especially visible in Fig. 3 A as the dots are more clearly visible and in the violin plot of Fig. 3D where we see that the gated model has both higher median error as well as more extreme errors. There was also a bit more variation in the different iterations of the model for the gated results, especially for age and MMSE with respectively three and two times higher standard deviation values as seen in Table 2. Beyond the significance of the individual models, 15 of 21 models showed lower rmse in the continuous model, even if slightly, a bias which has a *p*-value of 0.04 based on the binomial distribution.

### Contributions of the different markers on the gated model’s results

**Fig. 4:**
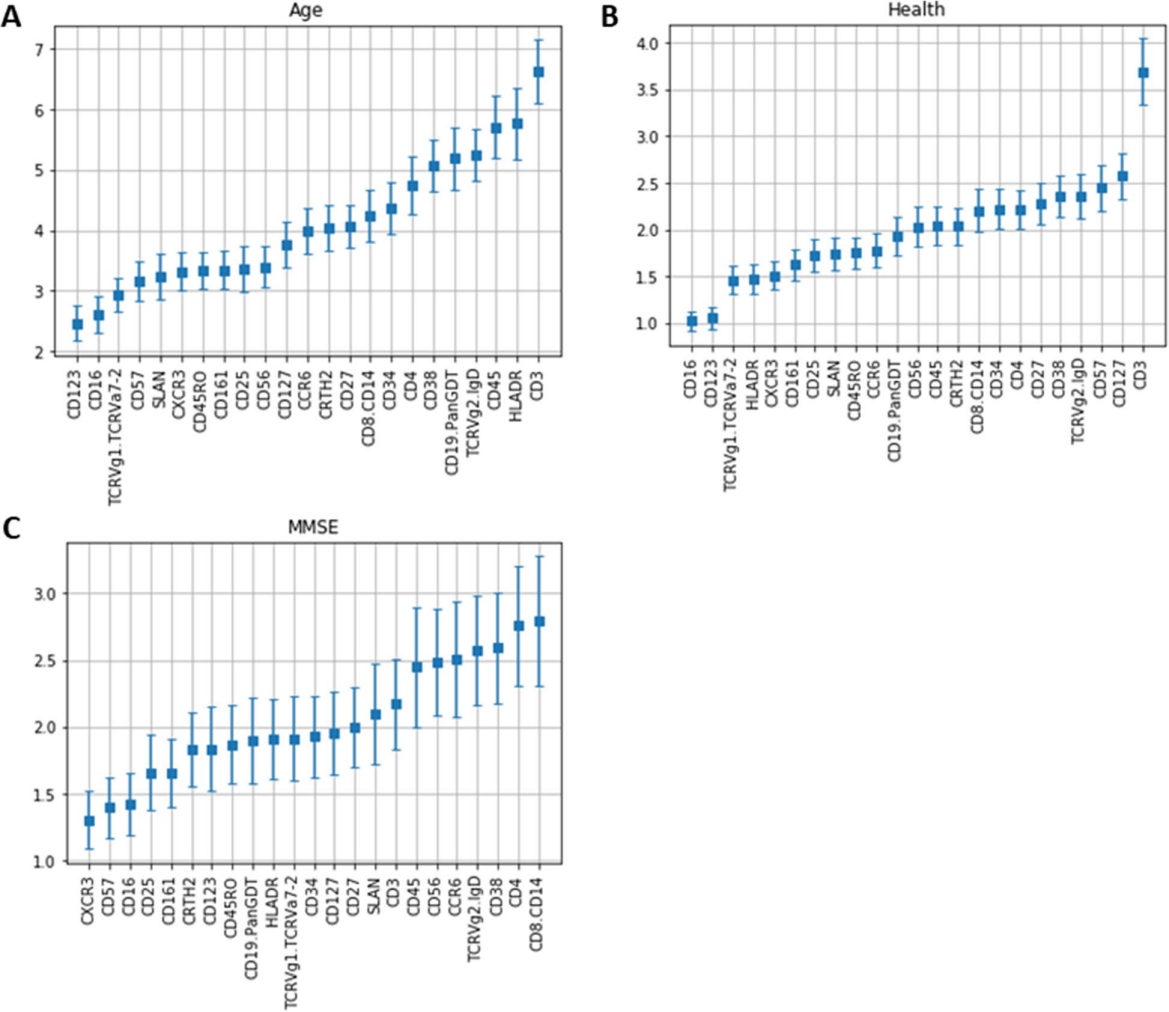
Values from the last layer of the non-gated model (informative layer) for successfully predicted outcomes, representing the contribution of that specific marker to the overall prediction. The middle square represents the mean value obtained for the 100 separate runs of the model and the bars are the standard error

We looked at the values added for each marker in the last layer of the non-gated model (informative layer, Fig. 1), because this value represents the contribution of that marker to the prediction. The results (Fig. 4) show us that CD3 contributed a lot to the prediction of age and health, but not that much in MMSE. CD16 contributed very little to all three health measures.

## Discussion

Here we have shown that it is possible to extract useful information from the levels of immune cell surface markers without consideration of specific cell types, and that this information could even outperform information extracted from traditional gating techniques. For most of the health measures tested here, neither of our models was able to give a meaningful prediction, a finding that was not unexpected. The fact that both models were able to predict the same health measures indicates that those that were successfully predicted are likely to be linked to the immune system and not just successful by chance.

The distributions of the surface markers observed in this study, consistent with those normally reported in traditional gating studies [36–38], indicate that many cannot easily be categorized as present or absent, or minimally that there is substantial variation in the quantities of the markers present even when there may be a distinct class of cells lacking the marker. This, together with our finding that a model built using raw surface marker abundance can outperform one built with traditional gating, points toward the potential of analyses considering cell surface marker abundance as a continuous rather than dichotomous measure. In our analysis, we noted certain markers as more or less important for the prediction of some health measures. CD3 was important for the prediction of age and health. It is a co-receptor that is used to identify T cells, indicating that this cell type might be important to determine these health outcomes. CD16 is present, among many other functionally similar receptors, on natural killer cells, monocytes, and macrophages and is implicated in the activation of those cells during an infection. Since it contributed very little to all three measures, it might mean that this function is not directly linked to these health outcomes or other markers of the innate immunity may be more specific. While CD3 is linked to adaptive immunity and CD16 is linked to innate immunity, this study could help discriminate the association of the two arms of the immune system in health outcomes during aging. Indeed, T cells, as the CD3 surface marker indicates, may be important player of the age-related changes in the immune response as well as of inflammaging. With aging the T cells may become senescent and acquire the Senescence associated secretory profile (SASP) and consequently secrete various pro-inflammatory mediators. These mediators will increase the senescence/exhaustion of T cells and contribute to the inflammaging. Inflammaging when goes uncontrolled may contribute to the traditionally considered age-related diseases such as cardiovascular diseases, cancer, neurodegenerative diseases. Results presented in Fig. 4 cannot be replicated for the gated model since it does not take raw surface markers abundance as an entry. This shows that building models using this kind of information can help discover more information about these markers in ways that might be difficult with a more traditional approach.

The approach shown here has several limitations. Most notably, while it appears that continuous information on surface marker abundance is relevant for understanding health, the approach used here does not consider which cells have which joint abundances of markers. Obviously, the relevance of a high abundance of a given marker on a cell may depend on the levels of the other markers on that same cell. It is analytically challenging to generate a portrait of an individual based on a composition of a large number of cells that are not discretely categorized and/or that vary along multiple axes (i.e., markers). Our goal was simply to show that the traditional gating approach implies a loss of relevant information. Second, despite the high-quality immune data available in SLAS via Flow Cytometry, we did not have the sample sizes needed to properly train models on specific immune pathologies or states (Table 1). The health measures we successfully predicted were all continuous or semi-continuous, suggesting that a lack of power was an important factor in the failure of other predictions. Third, this study was not designed to develop predictive models of health based on Flow Cytometry data, and accordingly, we make no claims about the power or relevance of the predictions made and attempted. Fourth, our analysis only includes people aged 55 and above. This limits the scope of our finding but does not exclude that our results might be generalizable to a broader population. Heterogeneity of the immune subpopulations increases during aging, making continuous analysis of the immune system even more suited for this type of population. Lastly, we note that the 27 surface markers included here are far from an exhaustive catalogue, and much more might be done with a more extensive list.

These are important limitations, and we stress that our key goal here was to briefly demonstrate the potential of new ways to consider the variability of the immune system that might be developed based on the incredible richness of Flow Cytometry data, possibly encouraging more studies to be made with this type of approach. The traditional gating approach clearly results in loss of important biological information. Improvements to gating based on Bayesian clustering [39] or other methods that reduce the dataset to counts of discrete cell types are likely to provide marginal but not massive improvements. Given the limited literature looking at the immune system without dividing it into cell types, this study might be useful to stimulate more research from that angle. We hope our approach will stimulate further thought on how to integrate continuous variation in surface marker abundance into future analyses.

## Supplementary Information

**Additional file 1.** Distributions of all surface markers used in this article.

**Additional file 2.** Gating strategy used to generate the data for the gated model.

**Additional file 3.** Flow cytometry antibodies information.
