# Supplementary figures and images for: Novel approach to analysis of the immune system using an ungated model of immune surface marker abundance to predict health outcomes
Source: Immun Ageing. 2022 Aug 4;19:35. doi: 10.1186/s12979-022-00291-y (PMC9351261; doi:10.1186/s12979-022-00291-y)

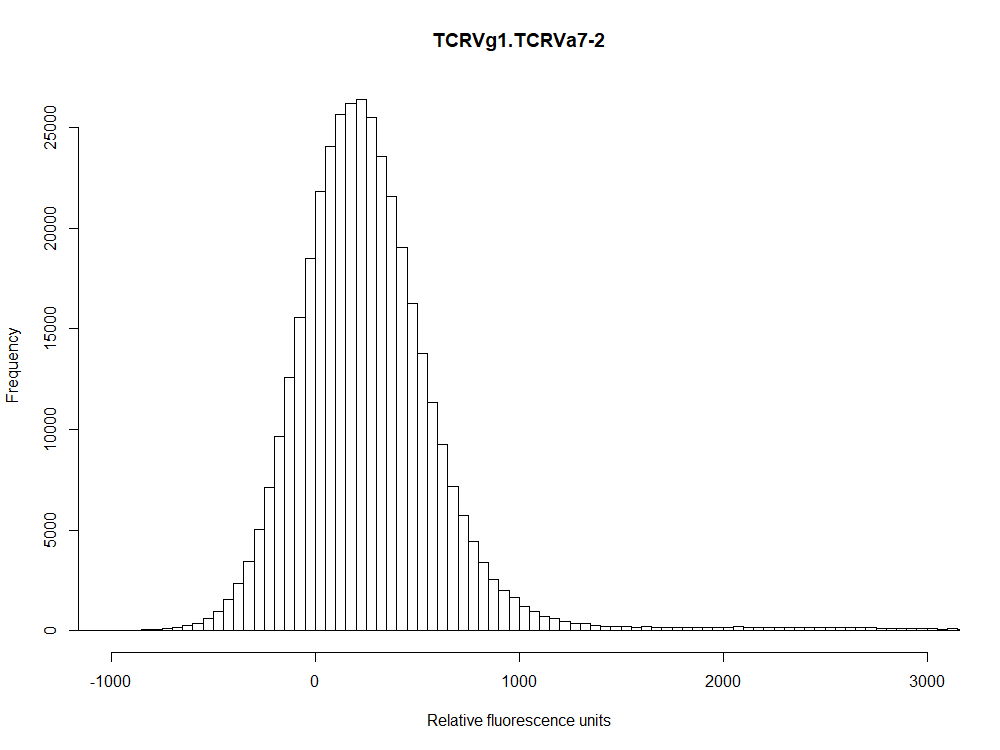

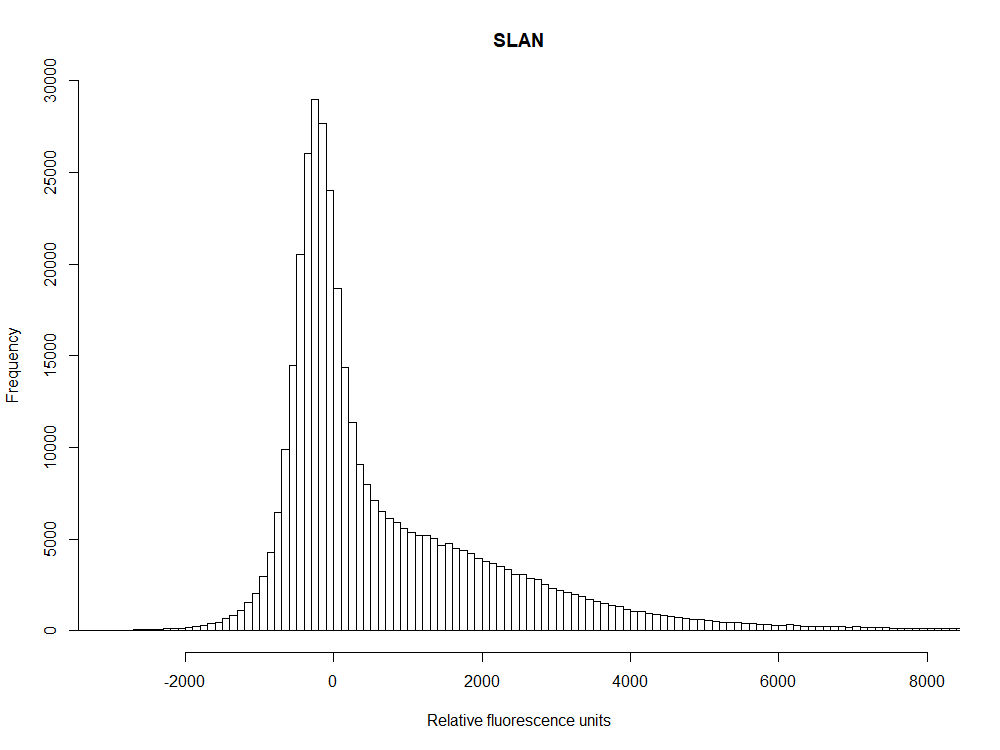

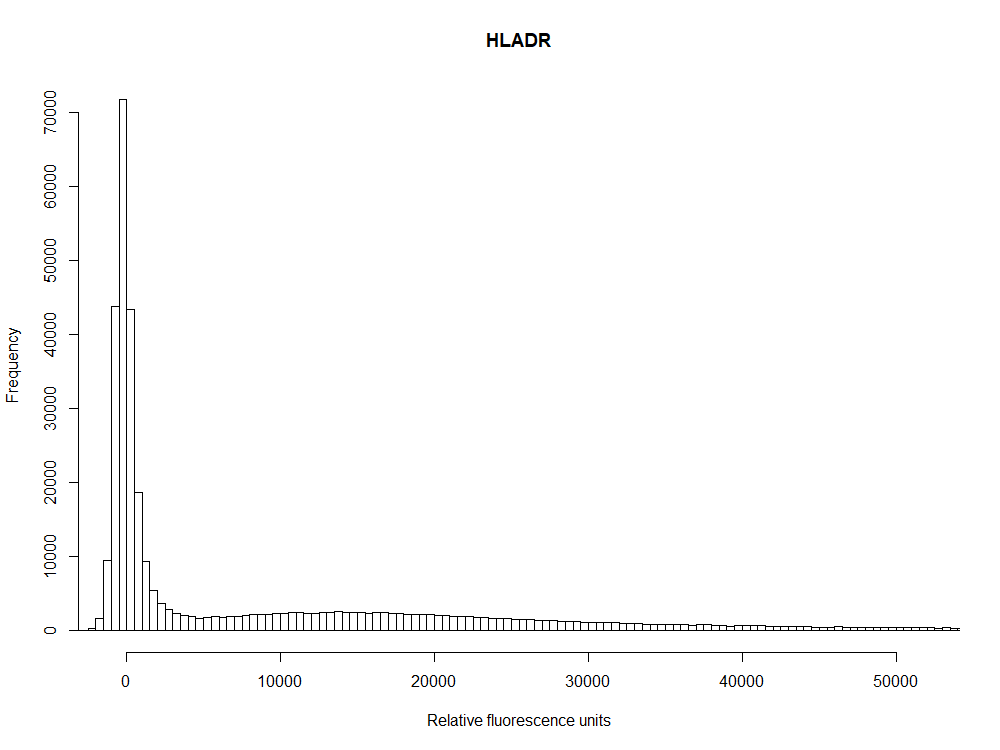

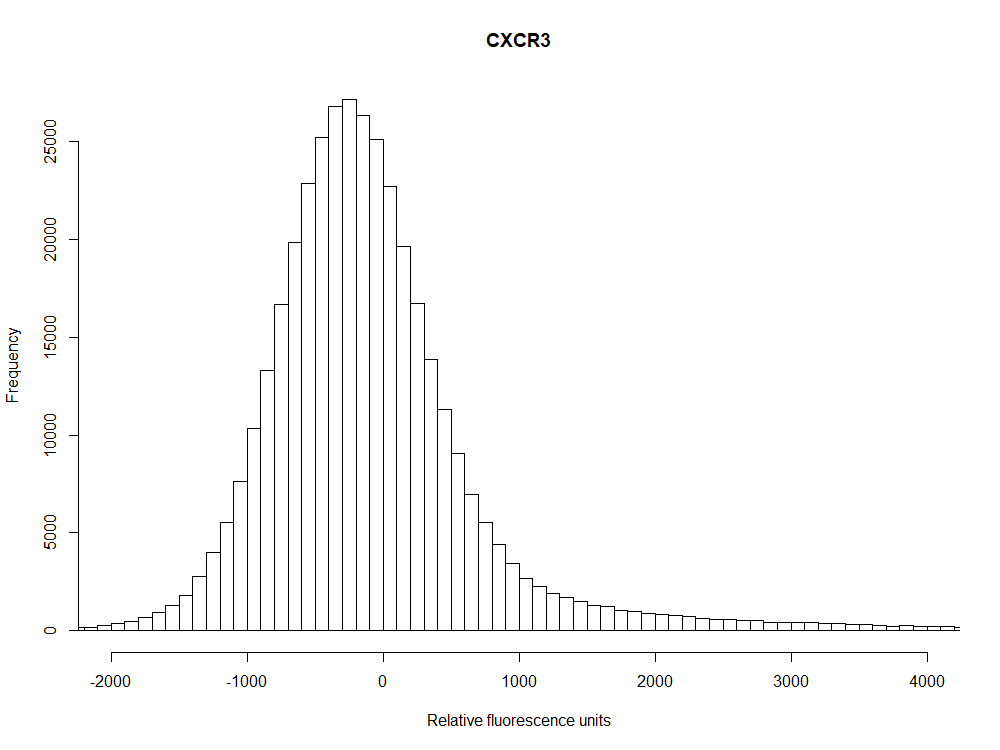

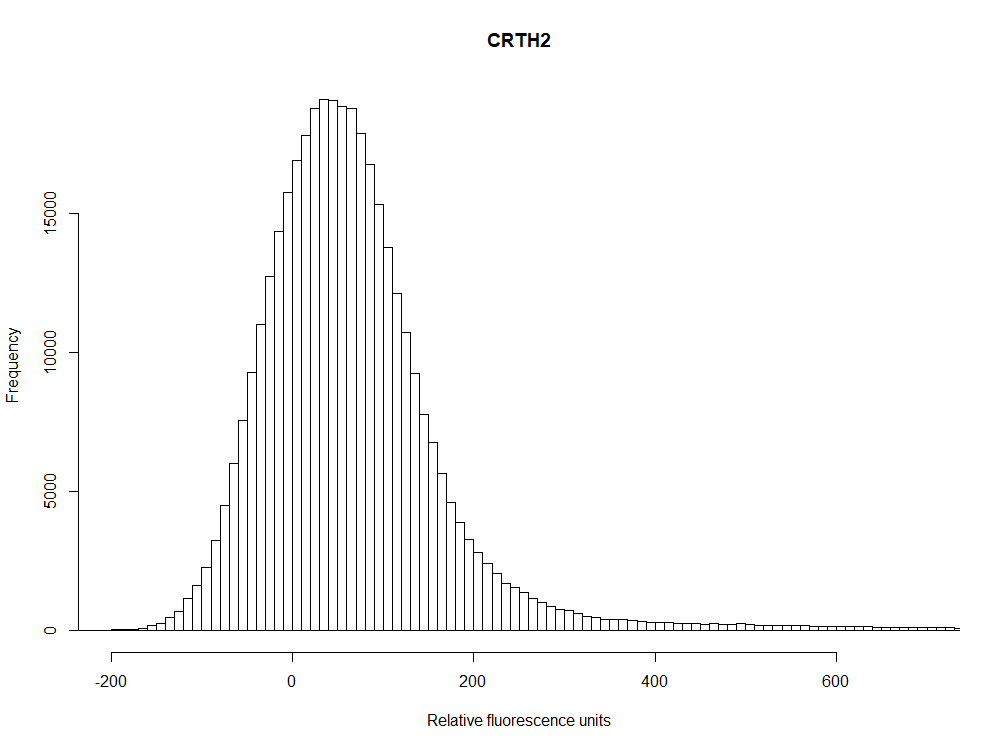

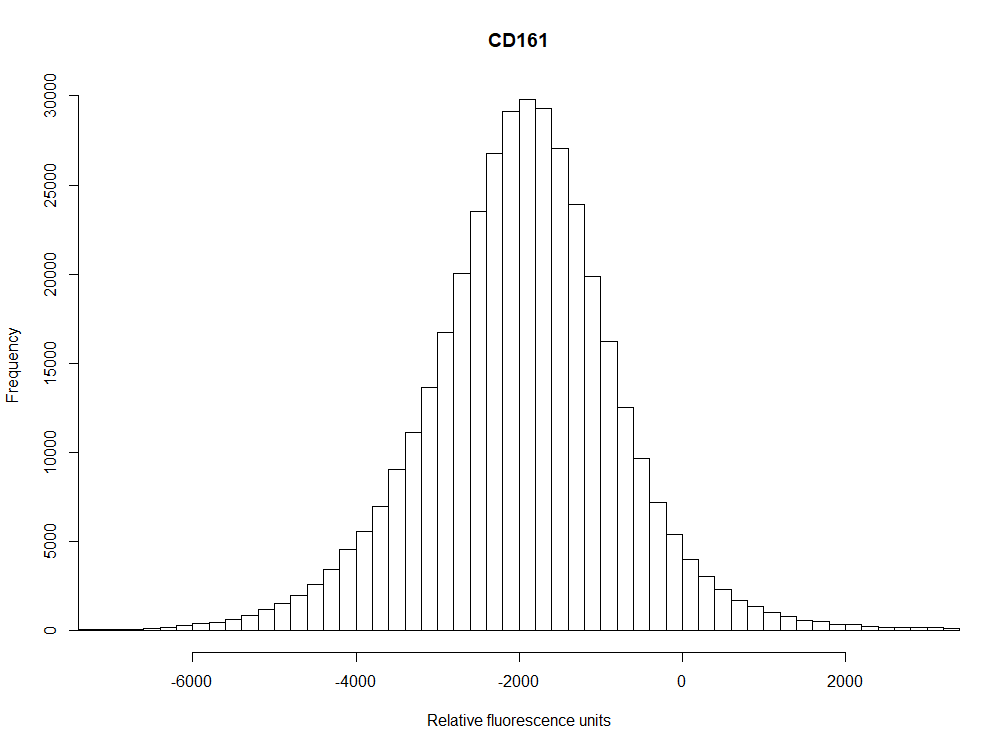

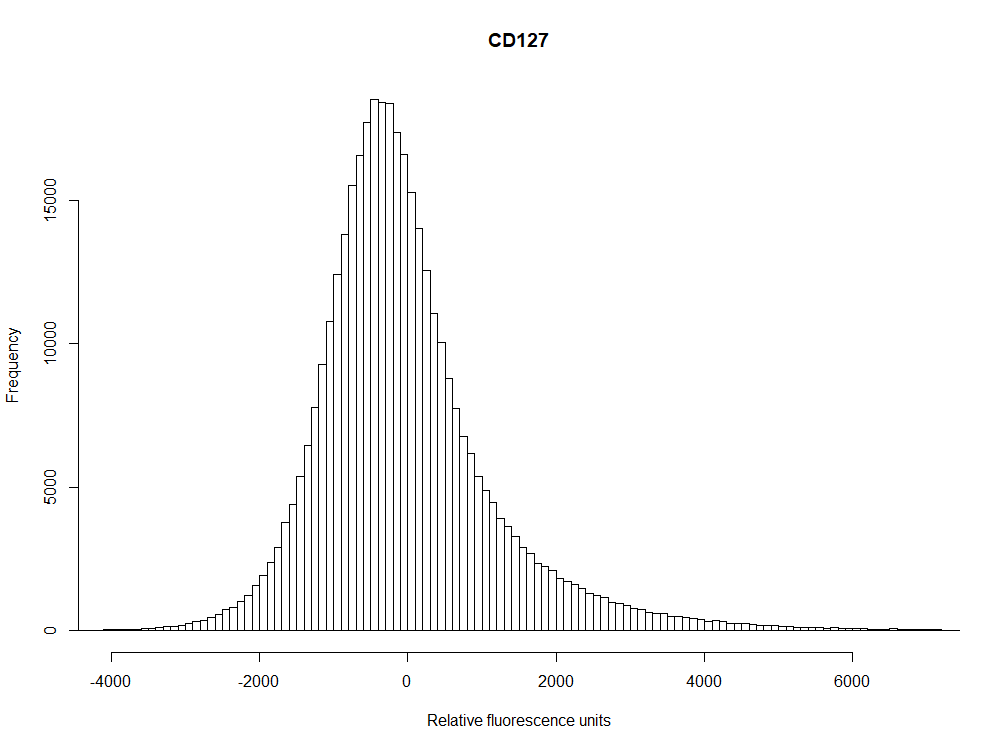

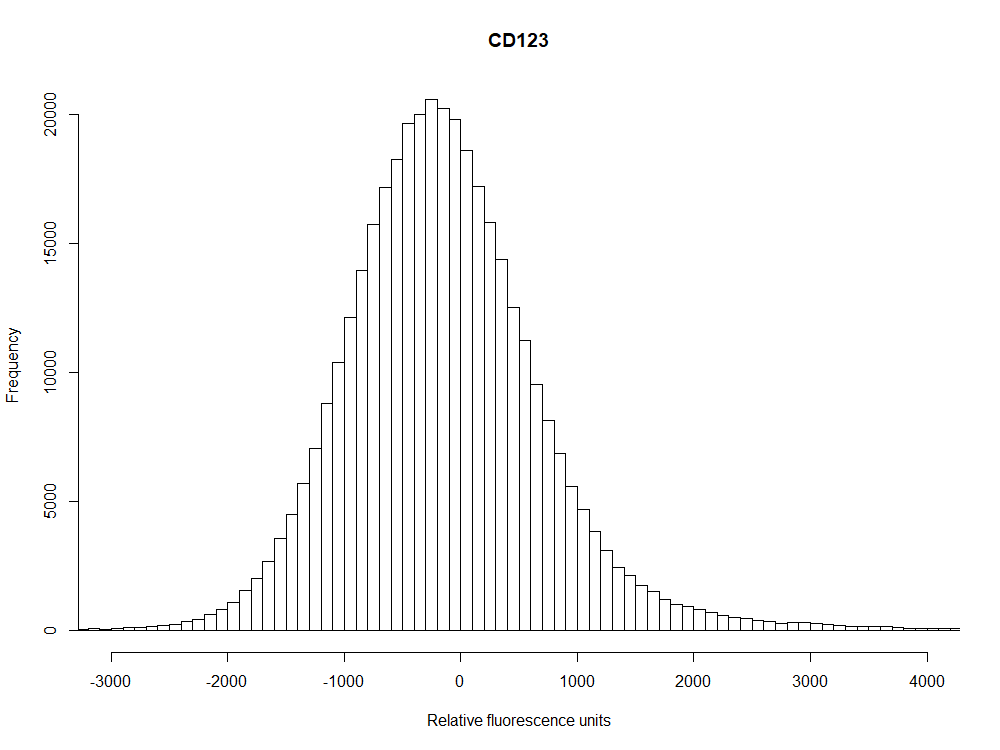

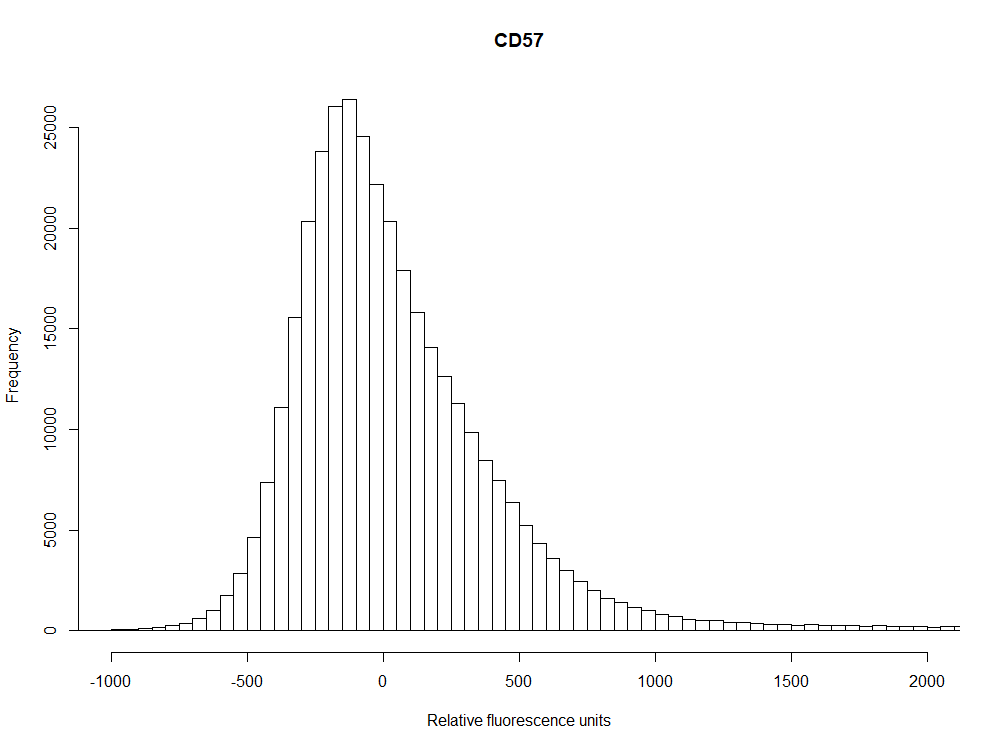

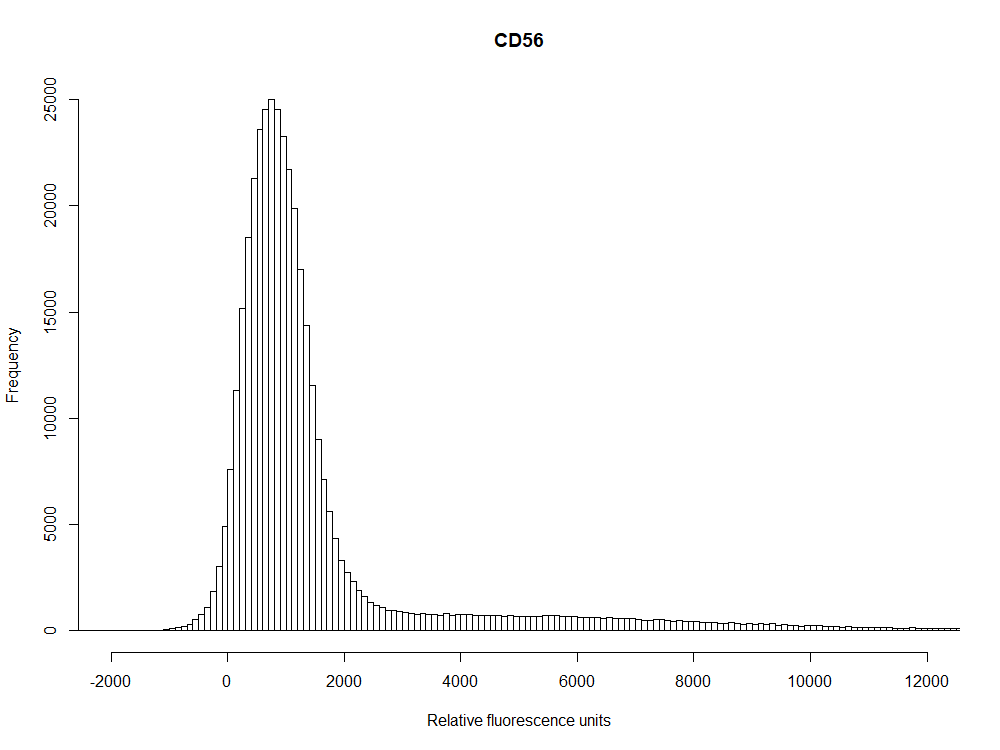

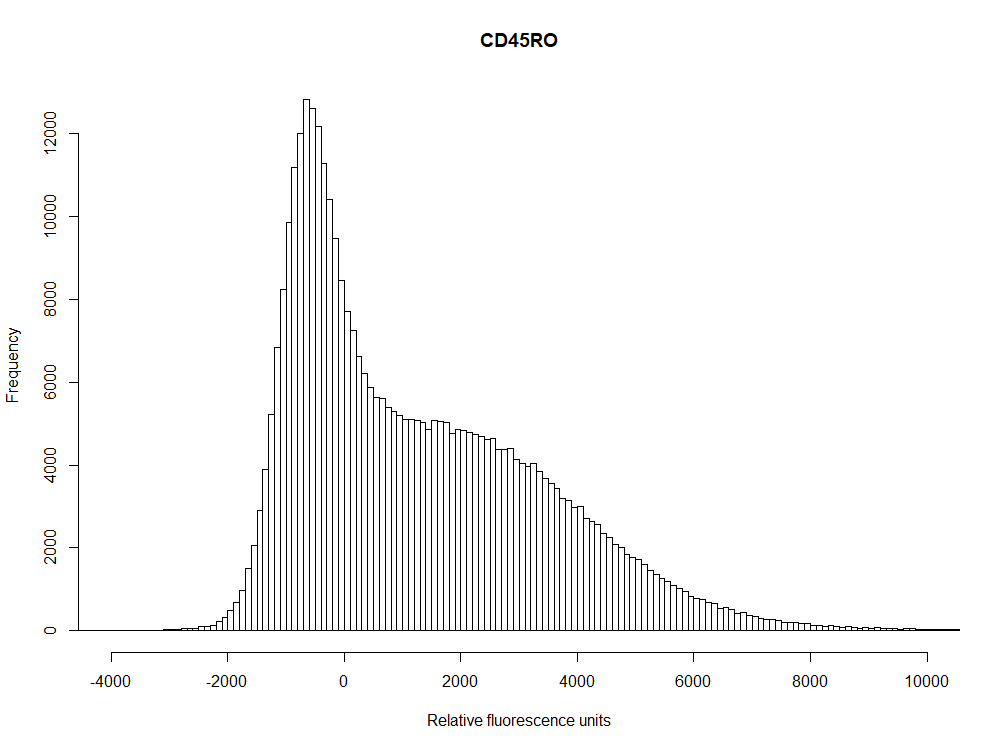

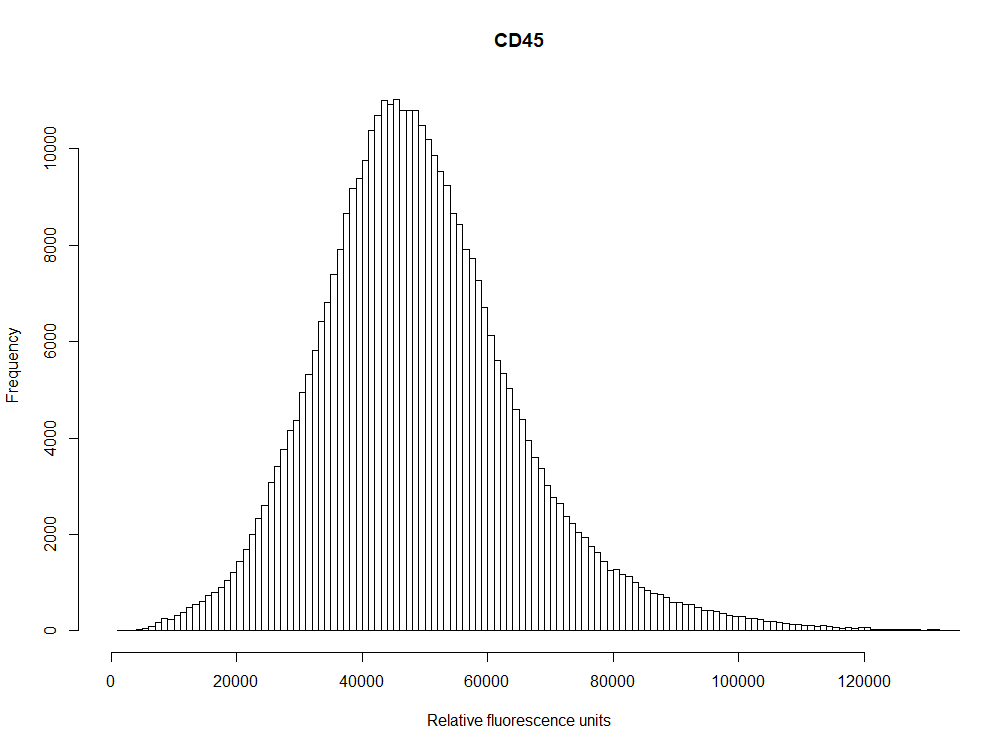

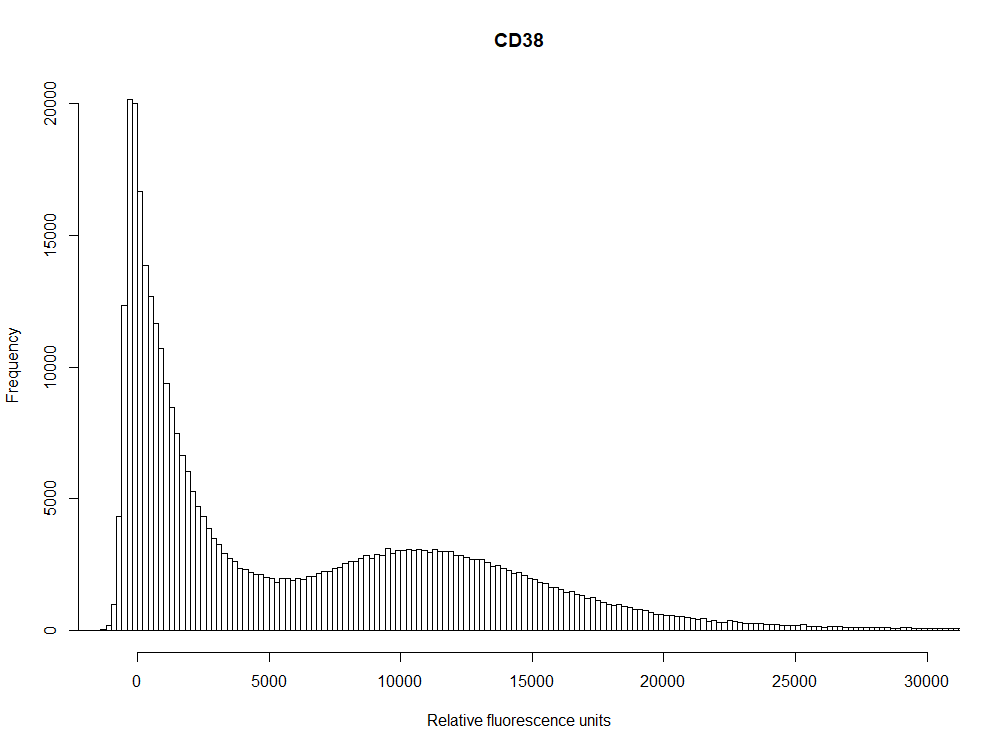

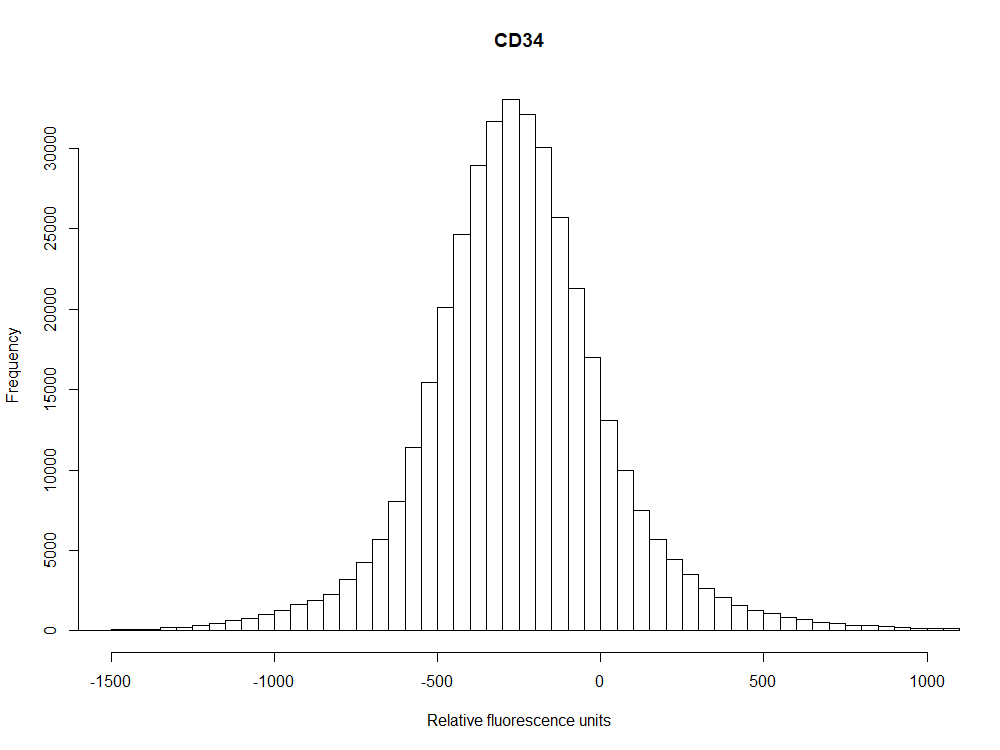

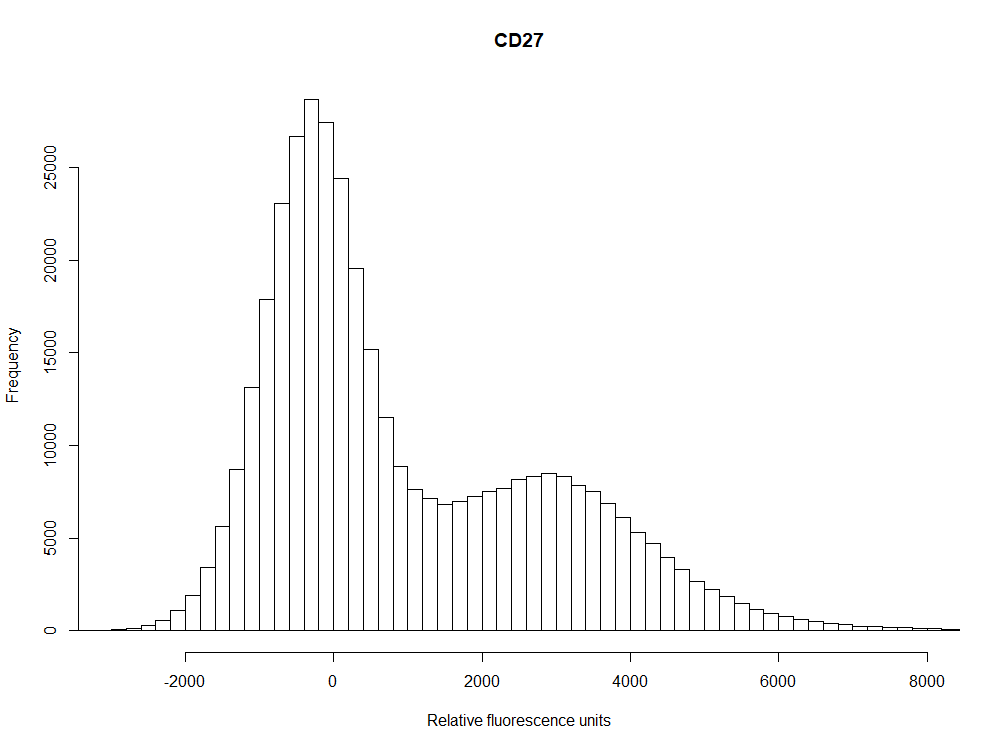

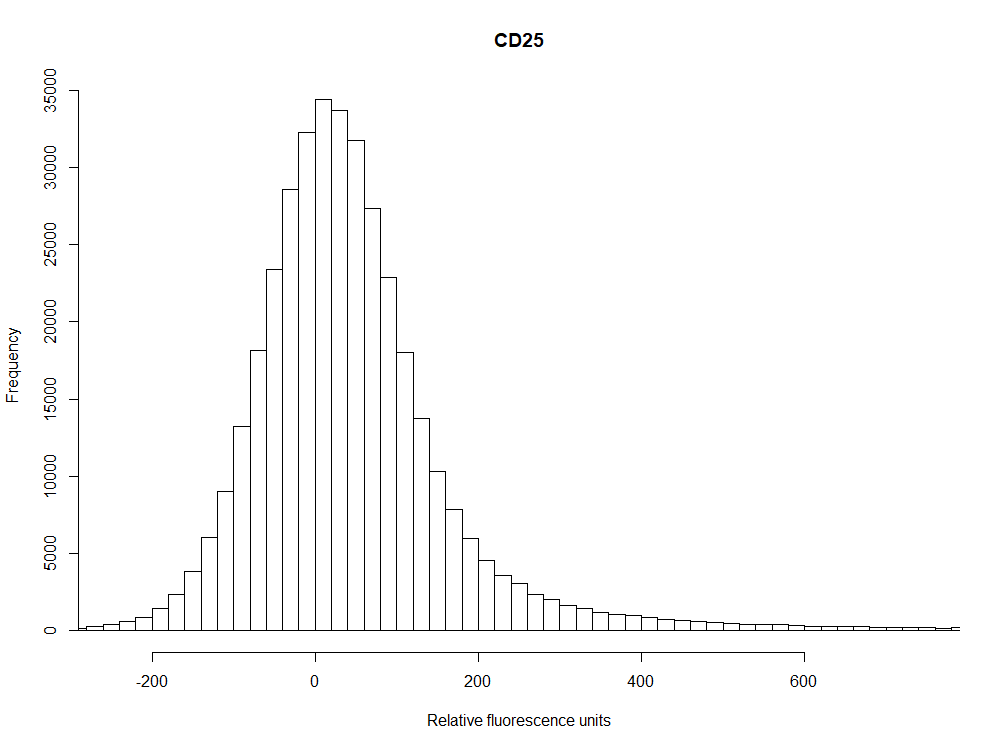

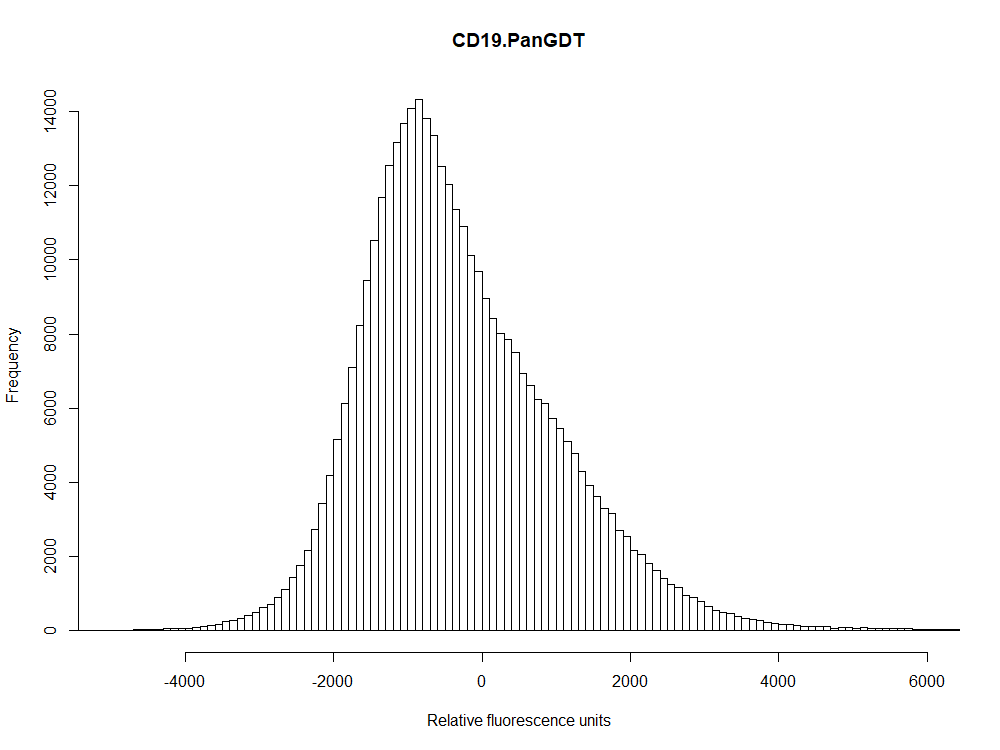

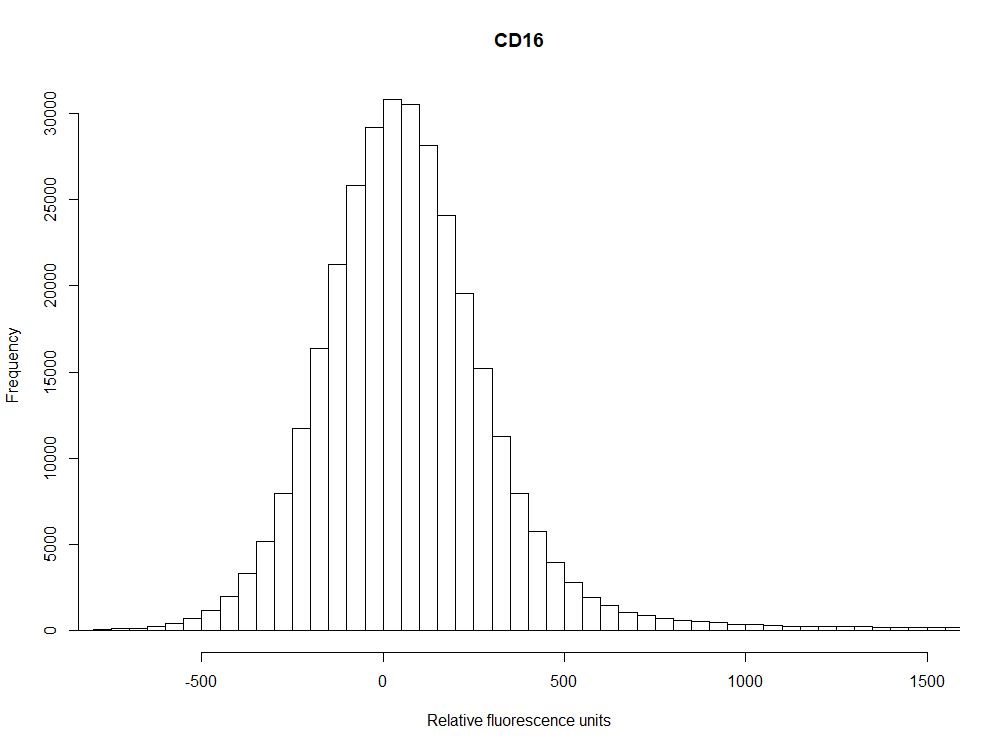

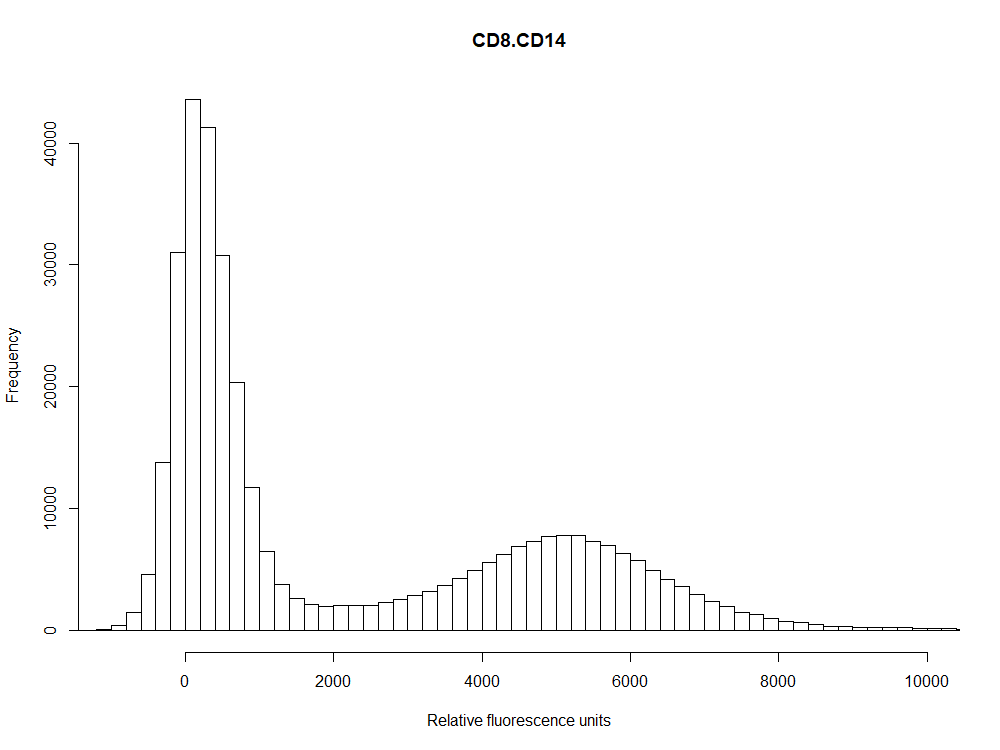

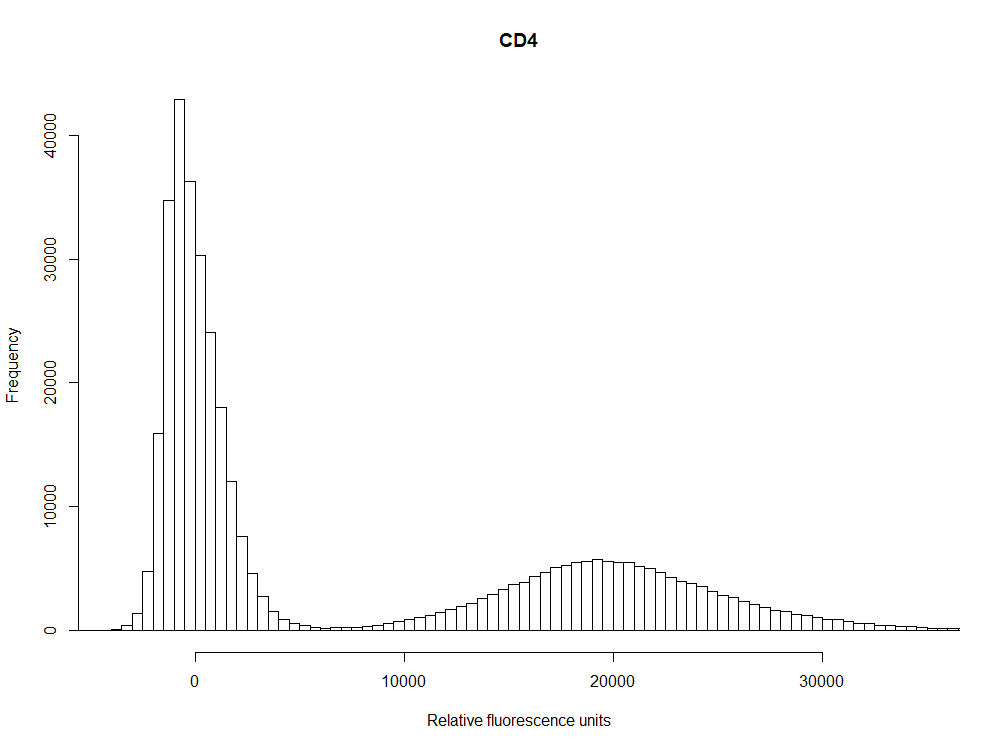

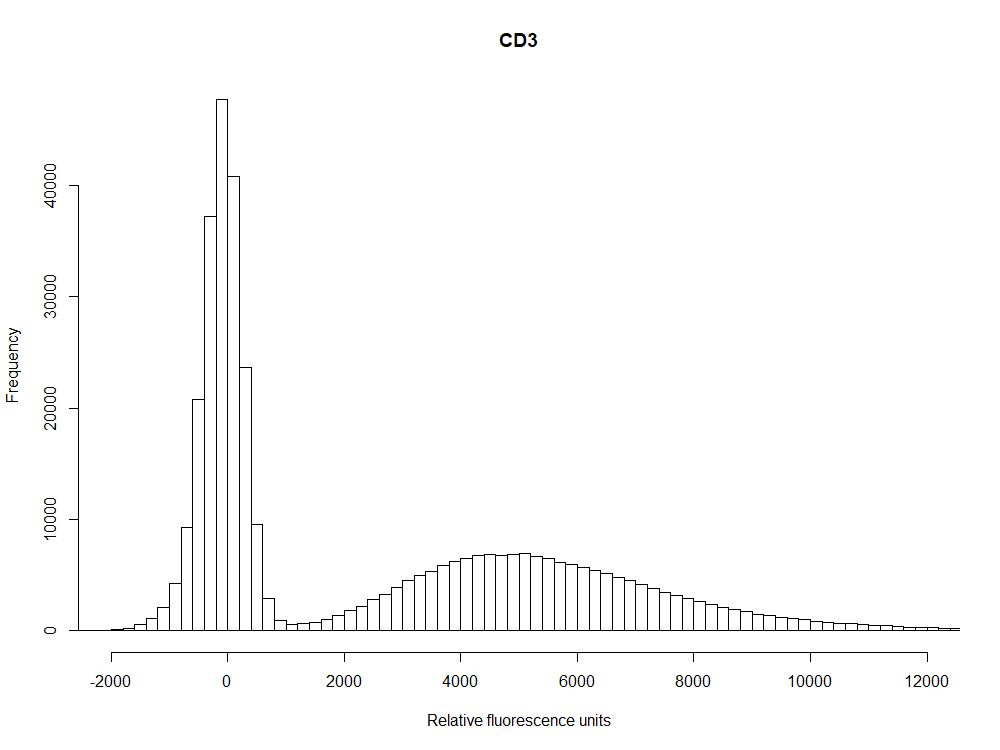

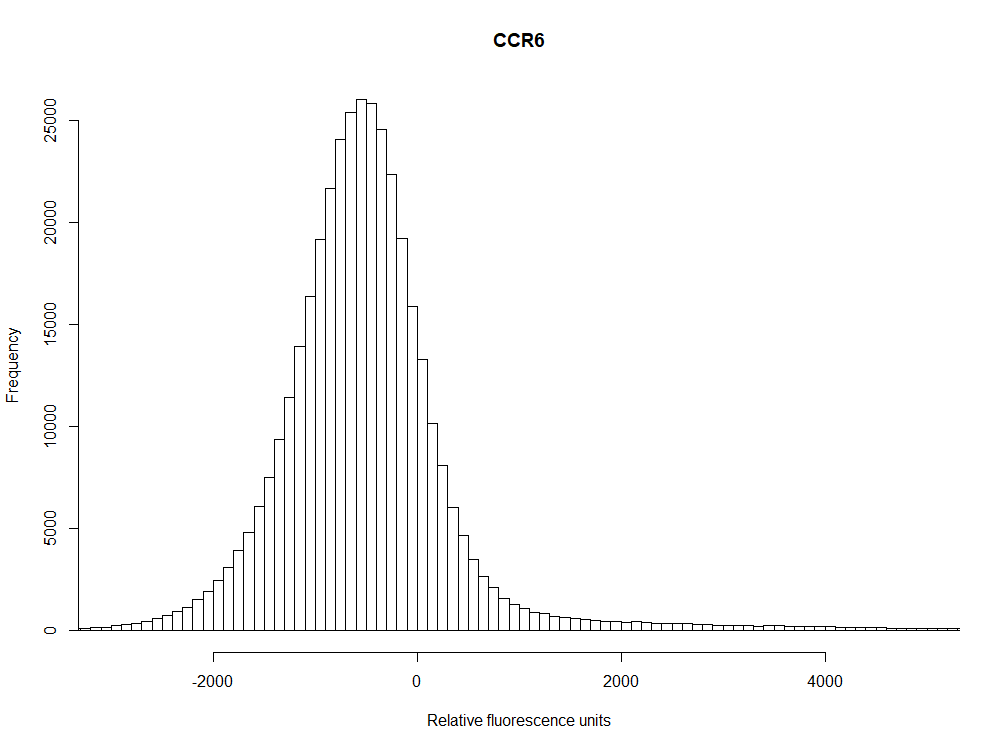

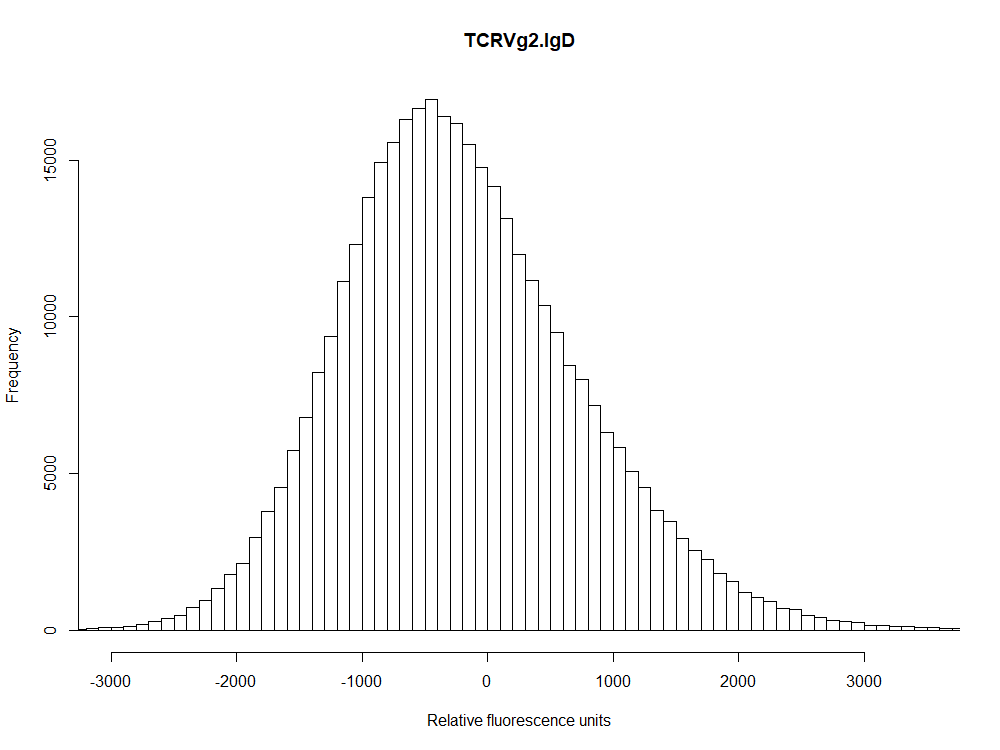

Supplement: Supplementary file 1 — Additional file 1. Distributions of all surface markers used in this article. [file 12979_2022_291_MOESM1_ESM.docx]
