# Supplementary material for: Novel approach to analysis of the immune system using an ungated model of immune surface marker abundance to predict health outcomes
Source: Immun Ageing. 2022 Aug 4;19:35. doi: 10.1186/s12979-022-00291-y (PMC9351261; doi:10.1186/s12979-022-00291-y)
Supplement: Supplementary file 2 — Additional file 2. Gating strategy used to generate the data for the gated model. [file 12979_2022_291_MOESM2_ESM.pdf]

Gating strategy tree

For SLAS cohort

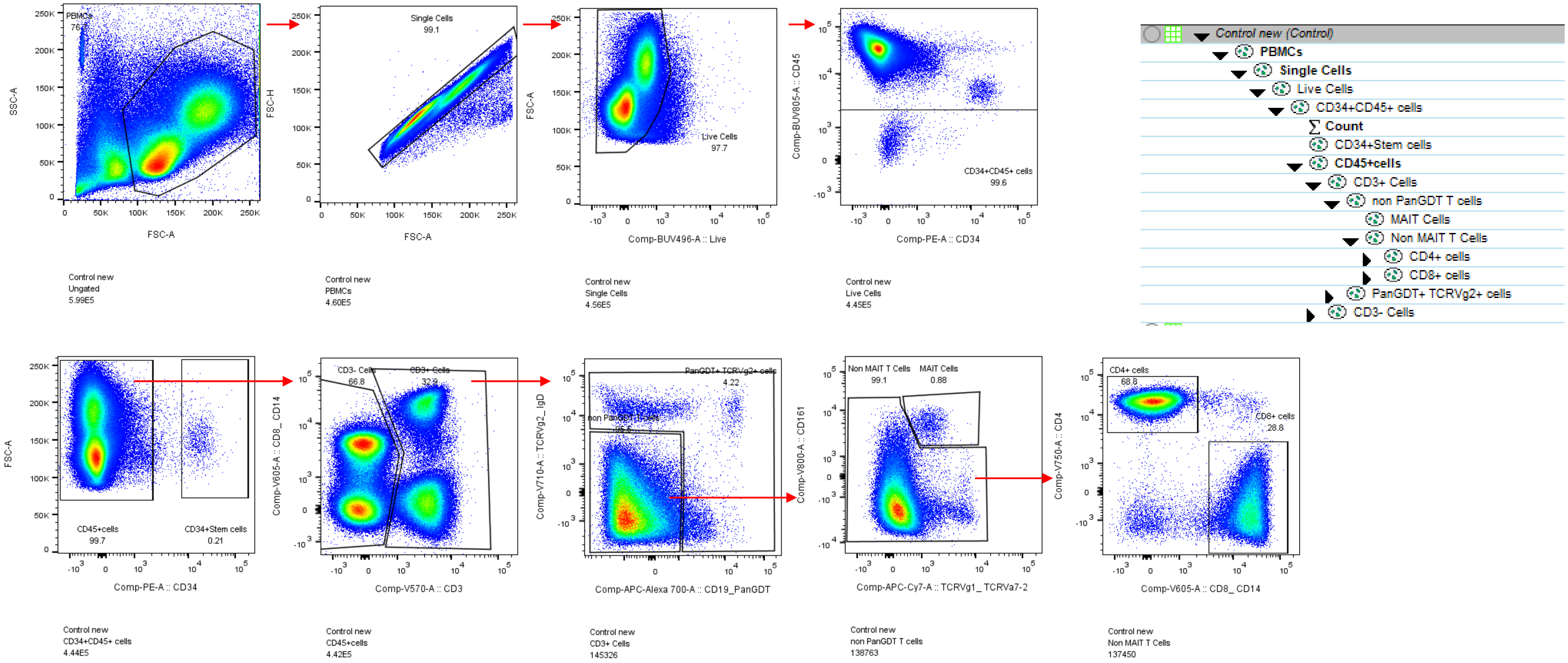

PBMCs
Single Cells
99.1
Live Cells
97.7
CD34+CD45+ cells
99.6
SSC-A
FSC-A
FSC-H
Comp-BUV496-A :: Live
Comp-BUV805-A :: CD45
Comp-PE-A :: CD34
Control new
Ungated
5.99E5
Control new
PBMCs
4.60E5
Control new
Single Cells
4.56E5
Control new
Live Cells
4.45E5
Control new (Control)
PBMCs
Single Cells
Live Cells
CD34+CD45+ cells
Count
CD34+Stem cells
CD45+cells
CD3+ Cells
non PanGDT T cells
MAIT Cells
Non MAIT T Cells
CD4+ cells
CD8+ cells
PanGDT+ TCRVg2+ cells
CD3- Cells
CD45+cells
99.7
CD34+Stem cells
0.21
CD3- Cells
66.8
CD3+ Cells
32.9
PanGDT+ TCRVg2+ cells
4.22
Non MAIT T Cells
99.1
MAIT Cells
0.88
CD4+ cells
68.8
CD8+ cells
28.8
Comp-V605-A :: CD8_ CD14
Comp-V570-A :: CD3
Comp-V710-A :: TCRVg2_ IgD
Comp-APC-Alexa 700-A :: CD19_PanGDT
Comp-V800-A :: CD161
Comp-APC-Cy7-A :: TCRVg1_ TCRVa7-2
Comp-V750-A :: CD4
Control new
CD34+CD45+ cells
4.44E5
Control new
CD45+cells
4.42E5
Control new
CD3+ Cells
145326
Control new
non PanGDT T cells
138763
Control new
Non MAIT T Cells
137450

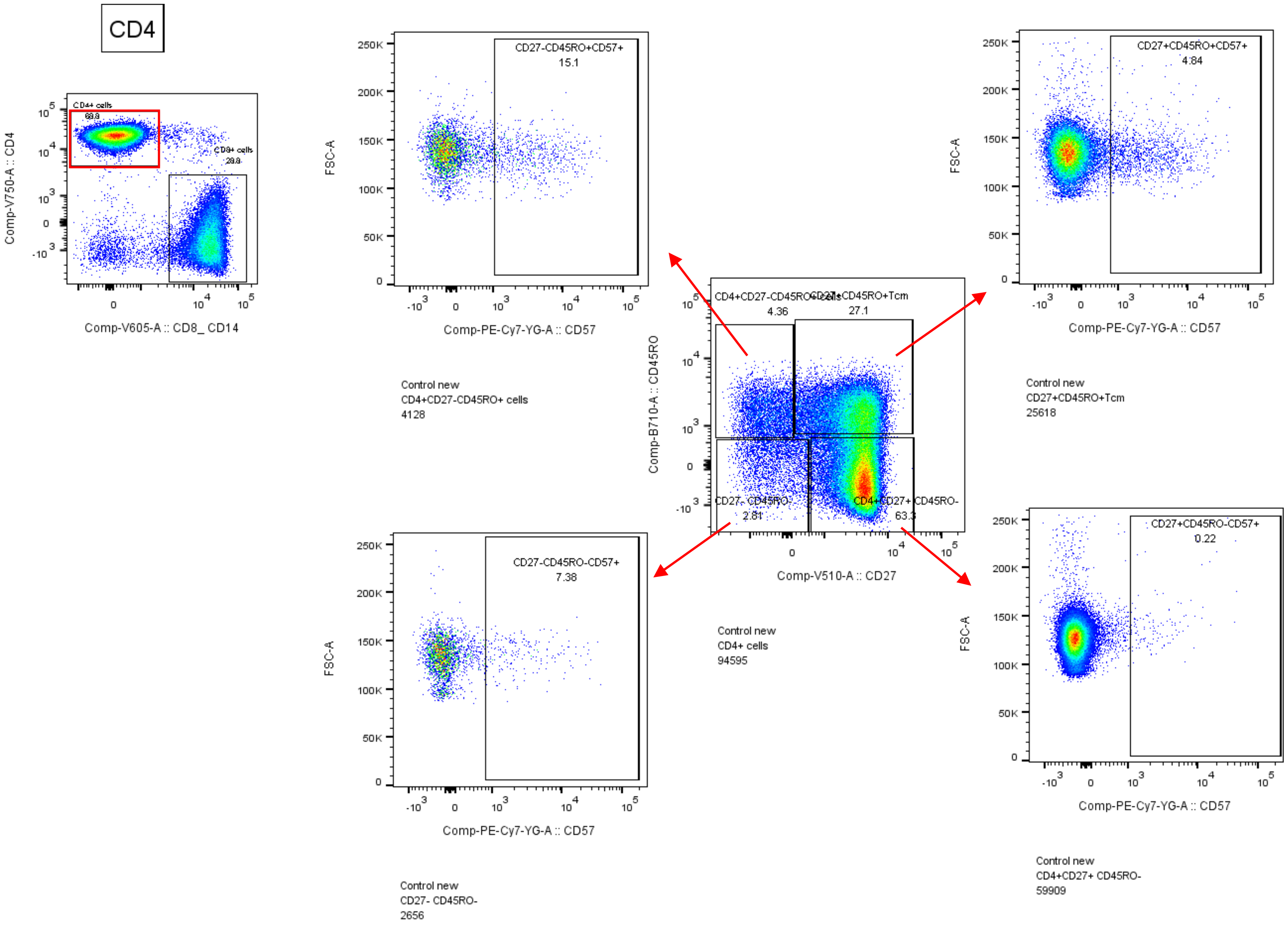
CD4
CD4+ cells
CD8+ cells
Comp-V750-A :: CD4
Comp-V605-A :: CD8_ CD14
CD27-CD45RO+CD57+
15.1
FSC-A
Comp-PE-Cy7-YG-A :: CD57
Control new
CD4+CD27-CD45RO+ cells
4128
CD27+CD45RO+CD57+
4.84
Control new
CD27+CD45RO+Tcm
25618
CD4+CD27-CD45RO+ cells
4.36
CD27+CD45RO+Tcm
27.1
CD27- CD45RO-
2.81
CD4+CD27+ CD45RO-
63.3
Comp-B710-A :: CD45RO
Comp-V510-A :: CD27
Control new
CD4+ cells
94595
CD27-CD45RO-CD57+
7.38
Control new
CD27- CD45RO-
2656
CD27+CD45RO-CD57+
0.22
Control new
CD4+CD27+ CD45RO-
59909

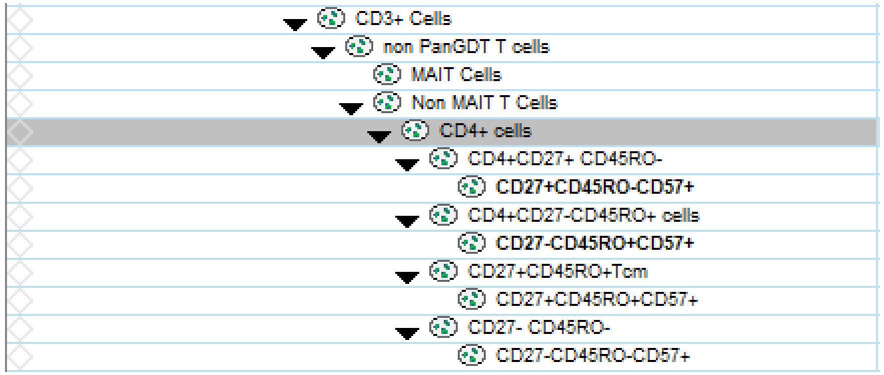
CD3+ Cells
non PanGDT T cells
MAIT Cells
Non MAIT T Cells
CD4+ cells
CD4+CD27+ CD45RO-
CD27+CD45RO-CD57+
CD4+CD27-CD45RO+ cells
CD27-CD45RO+CD57+
CD27+CD45RO+Tcm
CD27+CD45RO+CD57+
CD27- CD45RO-
CD27-CD45RO-CD57+

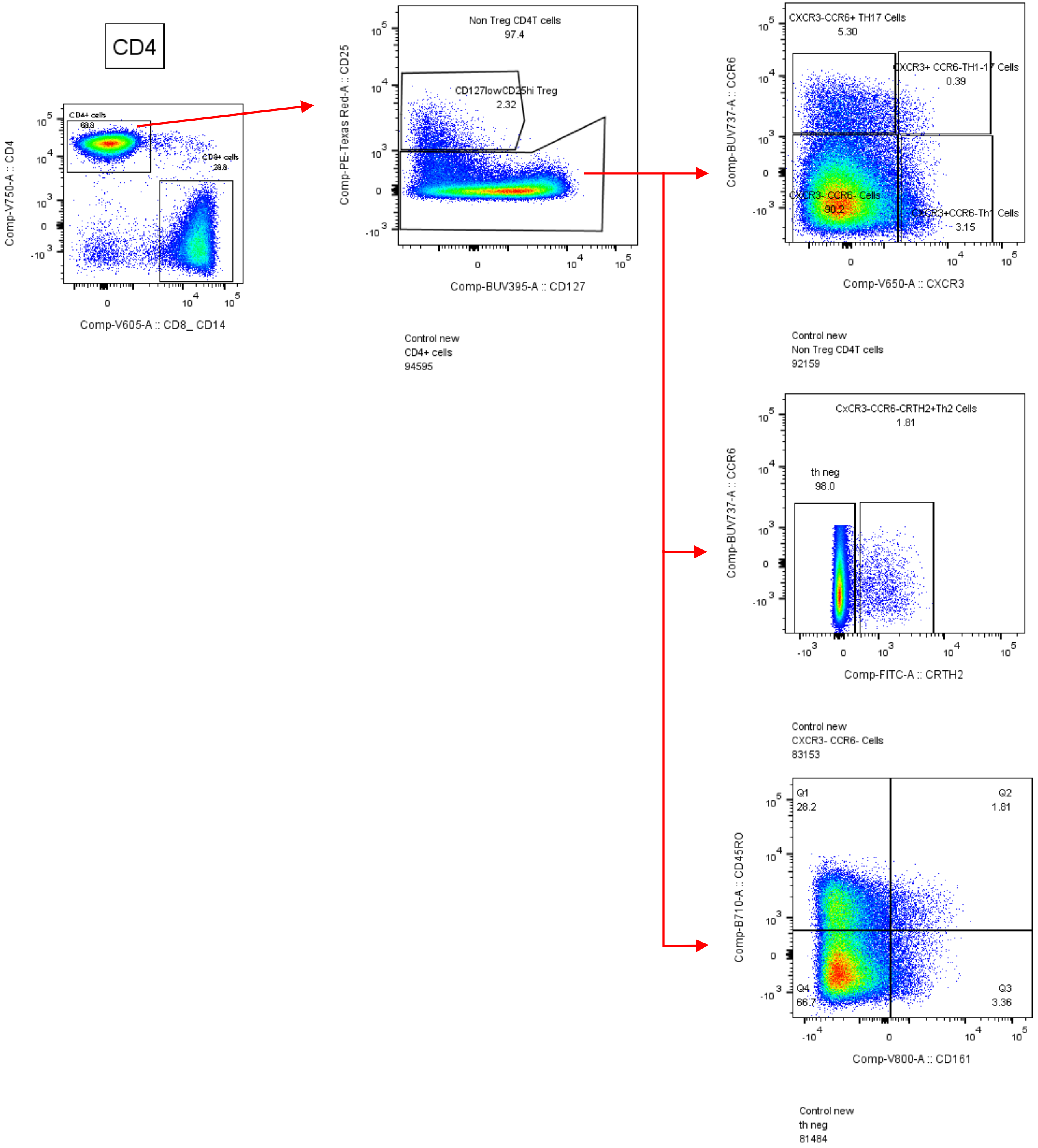

- CD3+ Cells
  - non PanGDT T cells
    - MAIT Cells
    - Non MAIT T Cells
      - CD4+ cells
        - CD4+CD27+ CD45RO-
          - **CD27+CD45RO-CD57+**
        - CD4+CD27-CD45RO+ cells
          - **CD27-CD45RO+CD57+**
        - CD27+CD45RO+Tcm
          - CD27+CD45RO+CD57+
        - CD27- CD45RO-
          - CD27-CD45RO-CD57+
        - **CD127lowCD25hi Treg**
        - **Non Treg CD4T cells**
          - CXCR3+ CCR6-TH1-17 Cells
          - CXCR3+CCR6-Th1 Cells
          - CXCR3- CCR6- Cells
            - CxCR3-CCR6-CRTH2+Th2 Cells
            - th neg
              - Q1: CD161- , CD45RO+
              - Q2: CD161+ , CD45RO+
              - Q3: CD161+ , CD45RO-
              - Q4: CD161- , CD45RO-
          - CXCR3-CCR6+ TH17 Cells

CD8

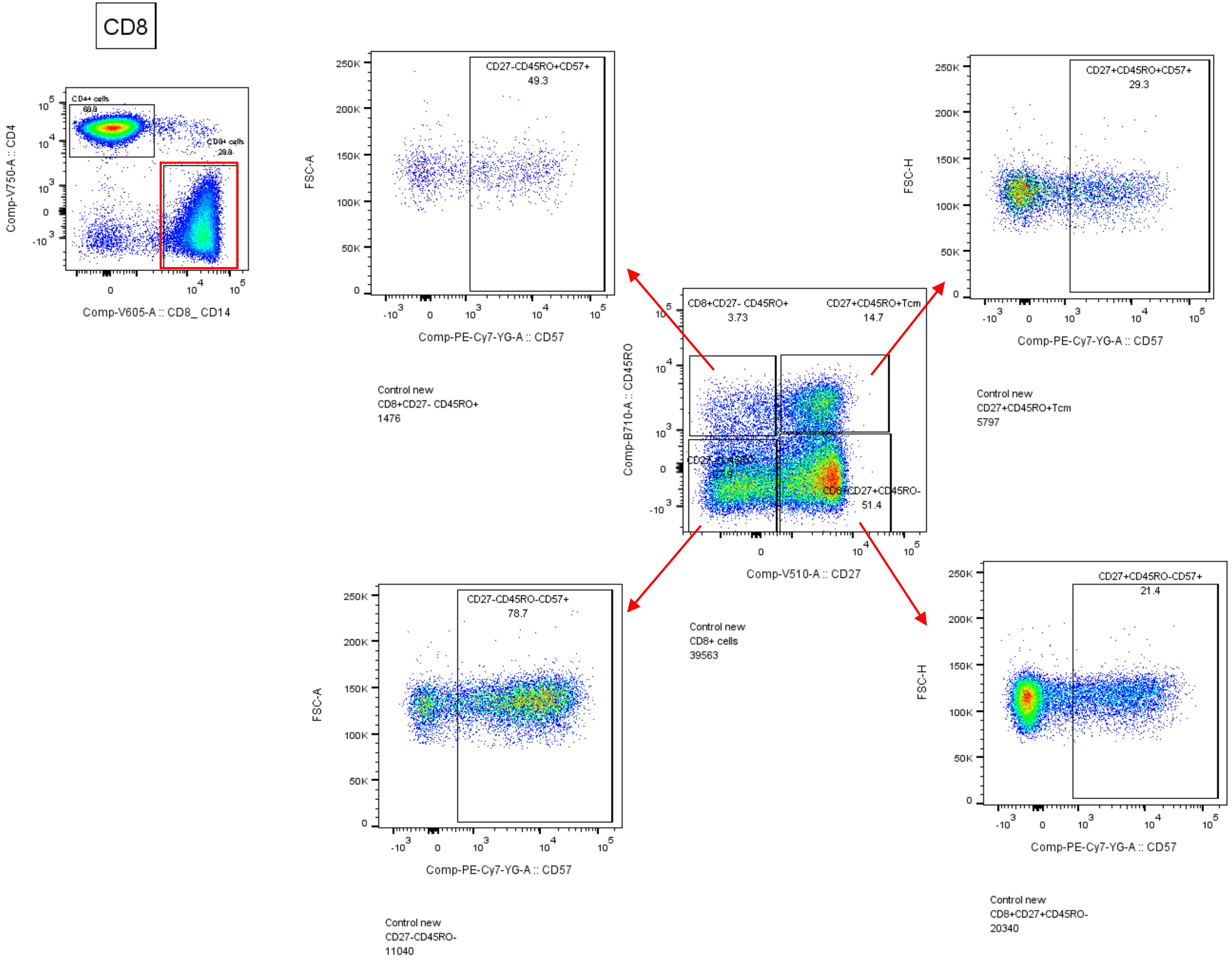
Comp-V750-A :: CD4
Comp-V605-A :: CD8_ CD14
CD4+ cells
CD8+ cells
CD27-CD45RO+CD57+
49.3
FSC-A
Comp-PE-Cy7-YG-A :: CD57
Control new
CD8+CD27- CD45RO+
1476
CD27+CD45RO+CD57+
29.3
FSC-H
Comp-PE-Cy7-YG-A :: CD57
Control new
CD27+CD45RO+Tcm
5797
CD8+CD27- CD45RO+
3.73
CD27+CD45RO+Tcm
14.7
CD8+CD27+CD45RO-
51.4
Comp-B710-A :: CD45RO
Comp-V510-A :: CD27
Control new
CD8+ cells
39563
CD27-CD45RO-CD57+
78.7
FSC-A
Comp-PE-Cy7-YG-A :: CD57
Control new
CD27-CD45RO-
11040
CD27+CD45RO-CD57+
21.4
FSC-H
Comp-PE-Cy7-YG-A :: CD57
Control new
CD8+CD27+CD45RO-
20340

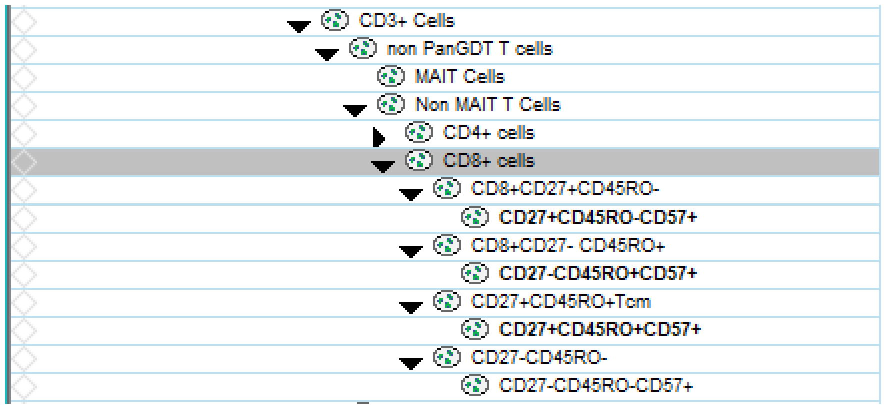
CD3+ Cells
non PanGDT T cells
MAIT Cells
Non MAIT T Cells
CD4+ cells
CD8+ cells
CD8+CD27+CD45RO-
CD27+CD45RO-CD57+
CD8+CD27- CD45RO+
CD27-CD45RO+CD57+
CD27+CD45RO+Tcm
CD27+CD45RO+CD57+
CD27-CD45RO-
CD27-CD45RO-CD57+

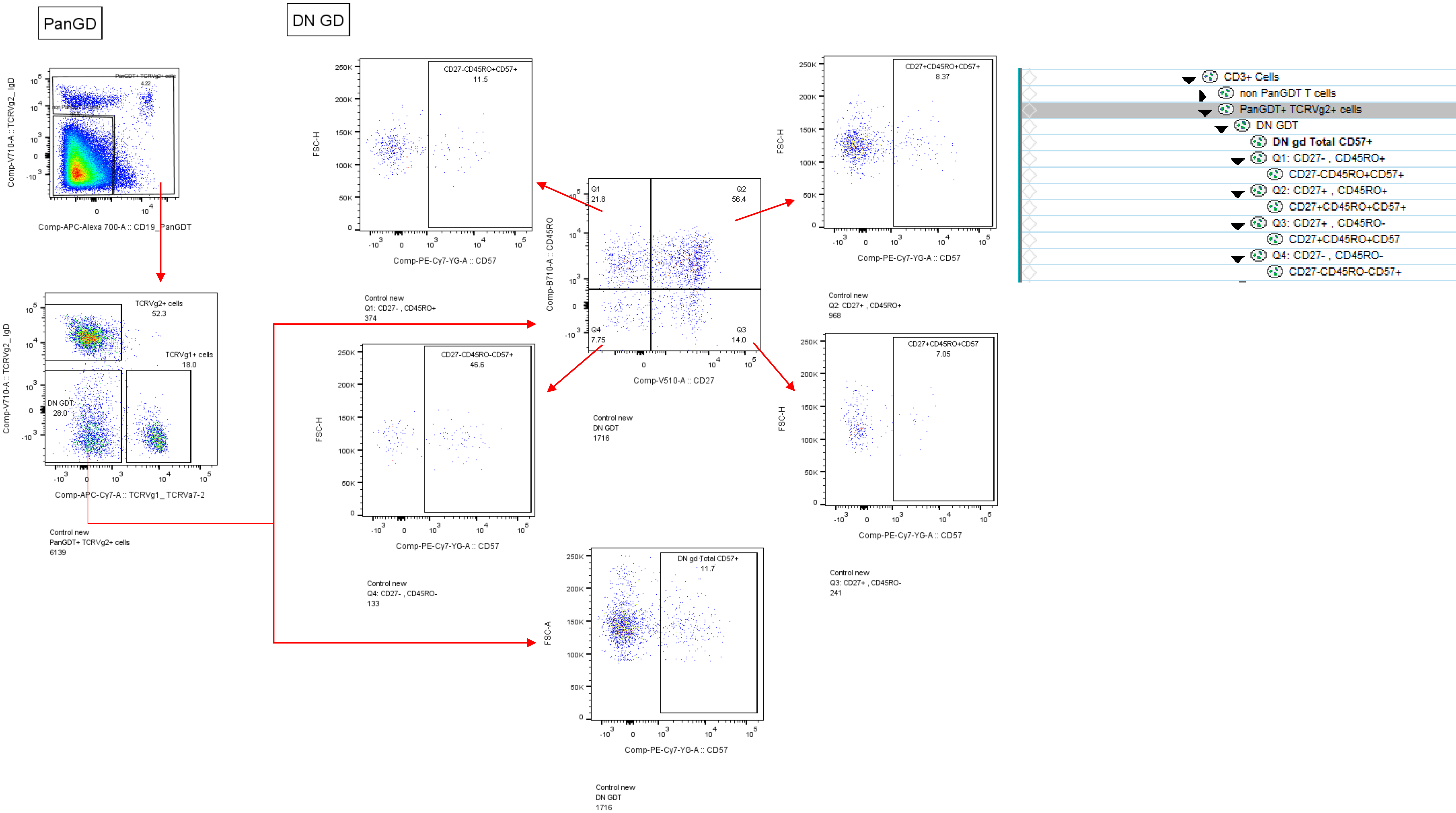

PanGD
DN GD
PanGDT+ TCRVg2+ cells
4.22
Comp-V710-A :: TCRVg2_IgD
Comp-APC-Alexa 700-A :: CD19_PanGDT
TCRVg2+ cells
52.3
TCRVg1+ cells
18.0
DN GDT
28.0
Comp-APC-Cy7-A :: TCRVg1_TCRVa7-2
Control new
PanGDT+ TCRVg2+ cells
6139
CD27-CD45RO+CD57+
11.5
FSC-H
Comp-PE-Cy7-YG-A :: CD57
Control new
Q1: CD27- , CD45RO+
374
CD27-CD45RO-CD57+
46.6
Control new
Q4: CD27- , CD45RO-
133
Q1
21.8
Q2
56.4
Q4
7.75
Q3
14.0
Comp-B710-A :: CD45RO
Comp-V510-A :: CD27
Control new
DN GDT
1716
DN gd Total CD57+
11.7
FSC-A
Control new
DN GDT
1716
CD27+CD45RO+CD57+
8.37
Control new
Q2: CD27+ , CD45RO+
968
CD27+CD45RO+CD57
7.05
Control new
Q3: CD27+ , CD45RO-
241
CD3+ Cells
non PanGDT T cells
PanGDT+ TCRVg2+ cells
DN GDT
DN gd Total CD57+
Q1: CD27- , CD45RO+
CD27-CD45RO+CD57+
Q2: CD27+ , CD45RO+
CD27+CD45RO+CD57+
Q3: CD27+ , CD45RO-
CD27+CD45RO+CD57
Q4: CD27- , CD45RO-
CD27-CD45RO-CD57+

Vd1

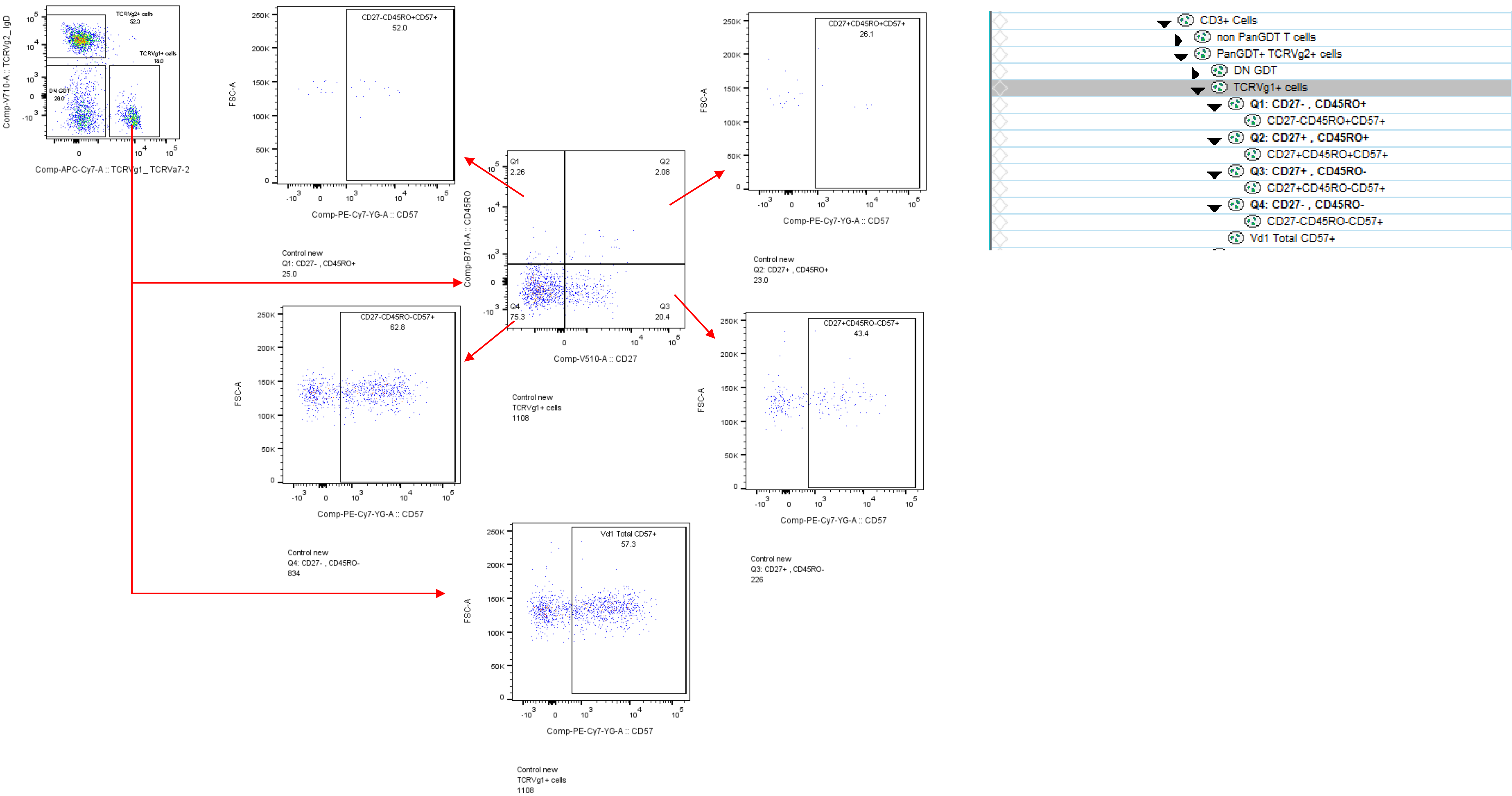

TCRVg2+ cells
52.0
TCRVg1+ cells
18.0
DN GDT
28.0
Comp-V710-A :: TCRVg2_ IgD
Comp-APC-Cy7-A :: TCRVg1_ TCRVa7-2
CD27-CD45RO+CD57+
52.0
FSC-A
Comp-PE-Cy7-YG-A :: CD57
Control new
Q1: CD27- , CD45RO+
25.0
Q1
2.26
Q2
2.08
Q4
75.3
Q3
20.4
Comp-B710-A :: CD45RO
Comp-V510-A :: CD27
Control new
TCRVg1+ cells
1108
CD27+CD45RO+CD57+
26.1
Control new
Q2: CD27+ , CD45RO+
23.0
CD27-CD45RO-CD57+
62.8
Control new
Q4: CD27- , CD45RO-
834
CD27+CD45RO-CD57+
43.4
Control new
Q3: CD27+ , CD45RO-
226
Vd1 Total CD57+
57.3
Control new
TCRVg1+ cells
1108
CD3+ Cells
non PanGDT T cells
PanGDT+ TCRVg2+ cells
DN GDT
TCRVg1+ cells
Q1: CD27- , CD45RO+
CD27-CD45RO+CD57+
Q2: CD27+ , CD45RO+
CD27+CD45RO+CD57+
Q3: CD27+ , CD45RO-
CD27+CD45RO-CD57+
Q4: CD27- , CD45RO-
CD27-CD45RO-CD57+
Vd1 Total CD57+

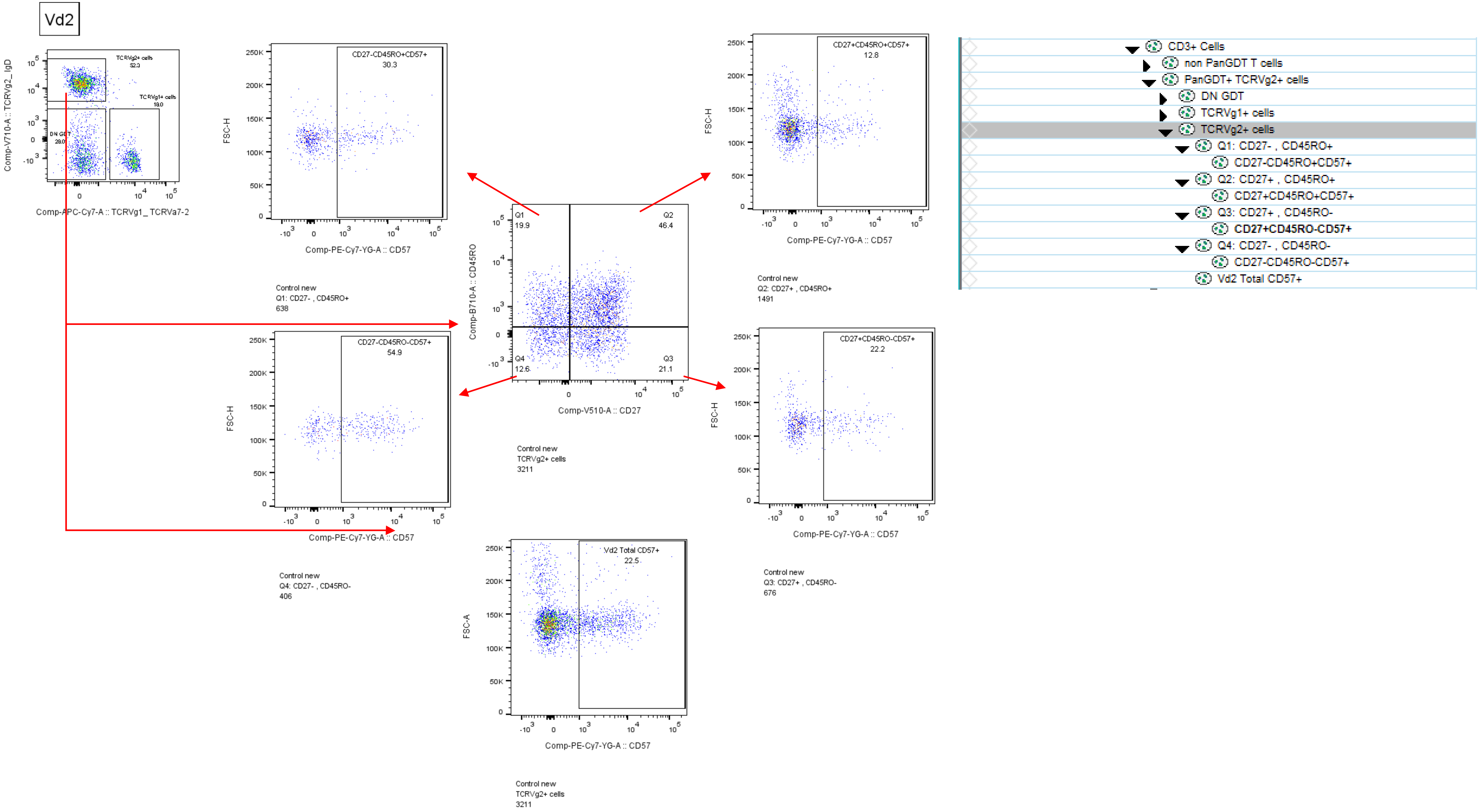
Vd2
Comp-V710-A :: TCRVg2_ IgD
Comp-APC-Cy7-A :: TCRVg1_ TCRVa7-2
TCRVg2+ cells
52.3
TCRVg1+ cells
18.0
DN GDT
28.0
CD27-CD45RO+CD57+
30.3
FSC-H
Comp-PE-Cy7-YG-A :: CD57
Control new
Q1: CD27- , CD45RO+
638
CD27+CD45RO+CD57+
12.8
Control new
Q2: CD27+ , CD45RO+
1491
Q1
19.9
Q2
46.4
Q4
12.6
Q3
21.1
Comp-B710-A :: CD45RO
Comp-V510-A :: CD27
Control new
TCRVg2+ cells
3211
CD27-CD45RO-CD57+
54.9
Control new
Q4: CD27- , CD45RO-
406
CD27+CD45RO-CD57+
22.2
Control new
Q3: CD27+ , CD45RO-
676
Vd2 Total CD57+
22.5
FSC-A
Control new
TCRVg2+ cells
3211
CD3+ Cells
non PanGDT T cells
PanGDT+ TCRVg2+ cells
DN GDT
TCRVg1+ cells
TCRVg2+ cells
Q1: CD27- , CD45RO+
CD27-CD45RO+CD57+
Q2: CD27+ , CD45RO+
CD27+CD45RO+CD57+
Q3: CD27+ , CD45RO-
CD27+CD45RO-CD57+
Q4: CD27- , CD45RO-
CD27-CD45RO-CD57+
Vd2 Total CD57+

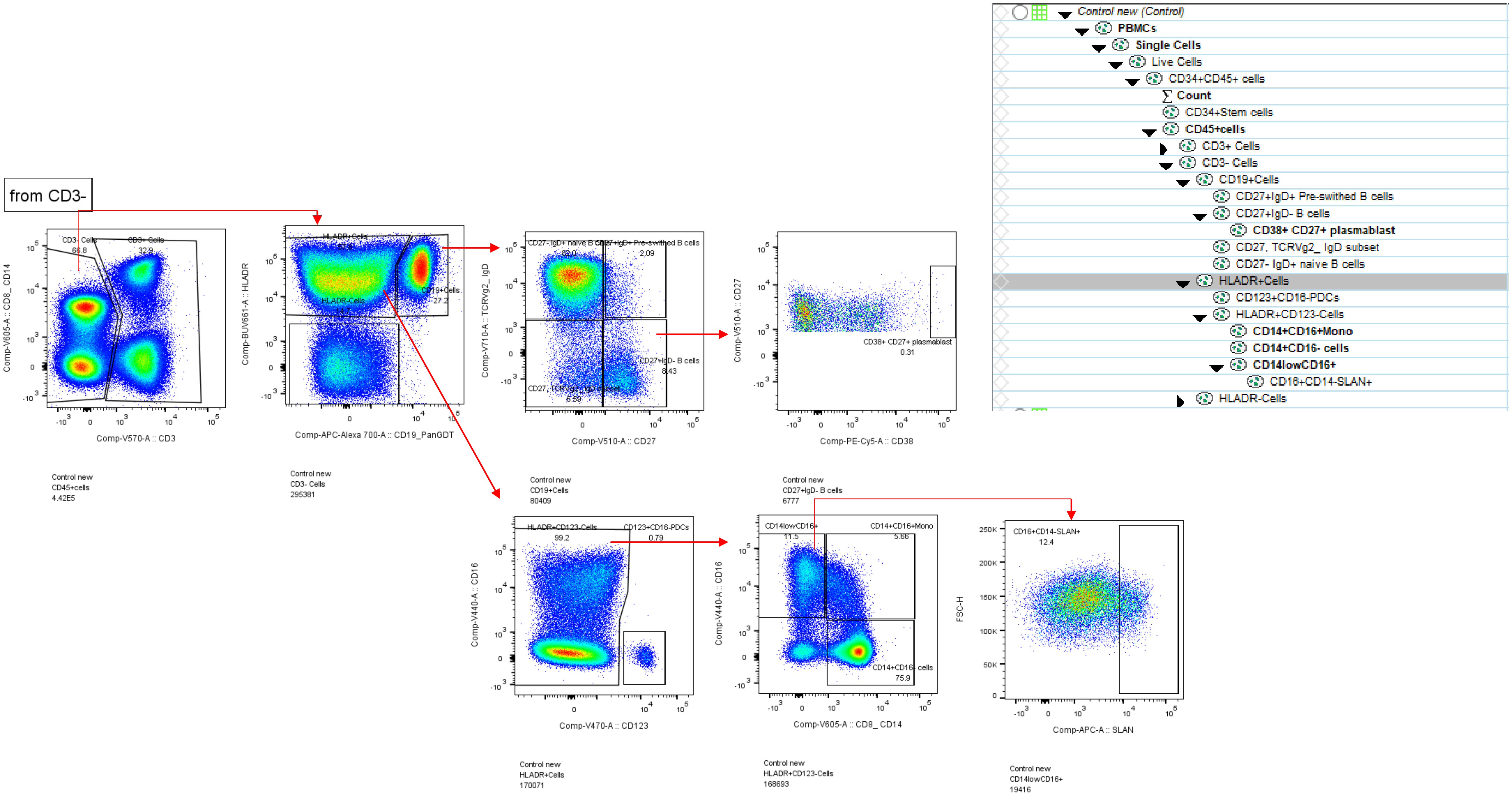

Control new (Control)
PBMCs
Single Cells
Live Cells
CD34+CD45+ cells
Count
CD34+Stem cells
CD45+cells
CD3+ Cells
CD3- Cells
CD19+Cells
CD27+IgD+ Pre-swithed B cells
CD27+IgD- B cells
CD38+ CD27+ plasmablast
CD27, TCRVg2_ IgD subset
CD27- IgD+ naive B cells
HLADR+Cells
CD123+CD16-PDCs
HLADR+CD123-Cells
CD14+CD16+Mono
CD14+CD16- cells
CD14lowCD16+
CD16+CD14-SLAN+
HLADR-Cells
from CD3-
CD3- Cells
66.8
CD3+ Cells
32.9
Comp-V605-A :: CD8_ CD14
Comp-V570-A :: CD3
Control new
CD45+cells
4.42E5
HLADR+Cells
HLADR-Cells
CD19+Cells
27.2
Comp-BUV661-A :: HLADR
Comp-APC-Alexa 700-A :: CD19_PanGDT
Control new
CD3- Cells
295381
CD27- IgD+ naive B cells
CD27+IgD+ Pre-swithed B cells
2.09
CD27+IgD- B cells
8.43
Comp-V710-A :: TCRVg2_ IgD
Comp-V510-A :: CD27
Control new
CD19+Cells
80409
CD38+ CD27+ plasmablast
0.31
Comp-V510-A :: CD27
Comp-PE-Cy5-A :: CD38
Control new
CD27+IgD- B cells
6777
HLADR+CD123-Cells
99.2
CD123+CD16-PDCs
0.79
Comp-V440-A :: CD16
Comp-V470-A :: CD123
Control new
HLADR+Cells
170071
CD14lowCD16+
11.5
CD14+CD16+Mono
5.66
CD14+CD16- cells
75.9
Comp-V440-A :: CD16
Comp-V605-A :: CD8_ CD14
Control new
HLADR+CD123-Cells
168693
CD16+CD14-SLAN+
12.4
FSC-H
Comp-APC-A :: SLAN
Control new
CD14lowCD16+
19416

HLADR-

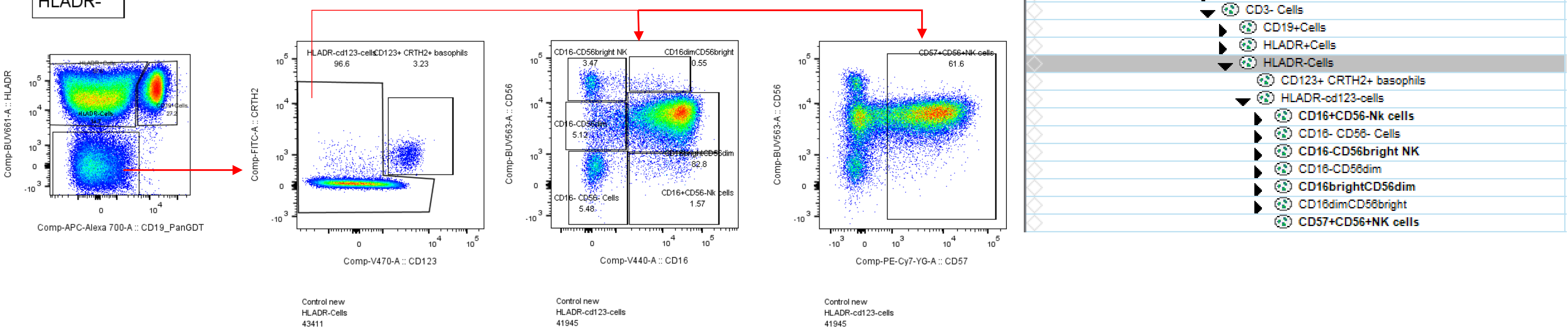

Comp-BUV661-A :: HLADR
Comp-APC-Alexa 700-A :: CD19_PanGDT
HLADR-cd123-cells
96.6
CD123+ CRTH2+ basophils
3.23
Comp-FITC-A :: CRTH2
Comp-V470-A :: CD123
Control new
HLADR-Cells
43411
CD16-CD56bright NK
3.47
CD16dimCD56bright
0.55
CD16-CD56dim
5.12
82.8
CD16- CD56- Cells
5.48
CD16+CD56-Nk cells
1.57
Comp-BUV563-A :: CD56
Comp-V440-A :: CD16
Control new
HLADR-cd123-cells
41945
CD57+CD56+NK cells
61.6
Comp-BUV563-A :: CD56
Comp-PE-Cy7-YG-A :: CD57
Control new
HLADR-cd123-cells
41945
CD3- Cells
CD19+Cells
HLADR+Cells
HLADR-Cells
CD123+ CRTH2+ basophils
HLADR-cd123-cells
CD16+CD56-Nk cells
CD16- CD56- Cells
CD16-CD56bright NK
CD16-CD56dim
CD16brightCD56dim
CD16dimCD56bright
CD57+CD56+NK cells

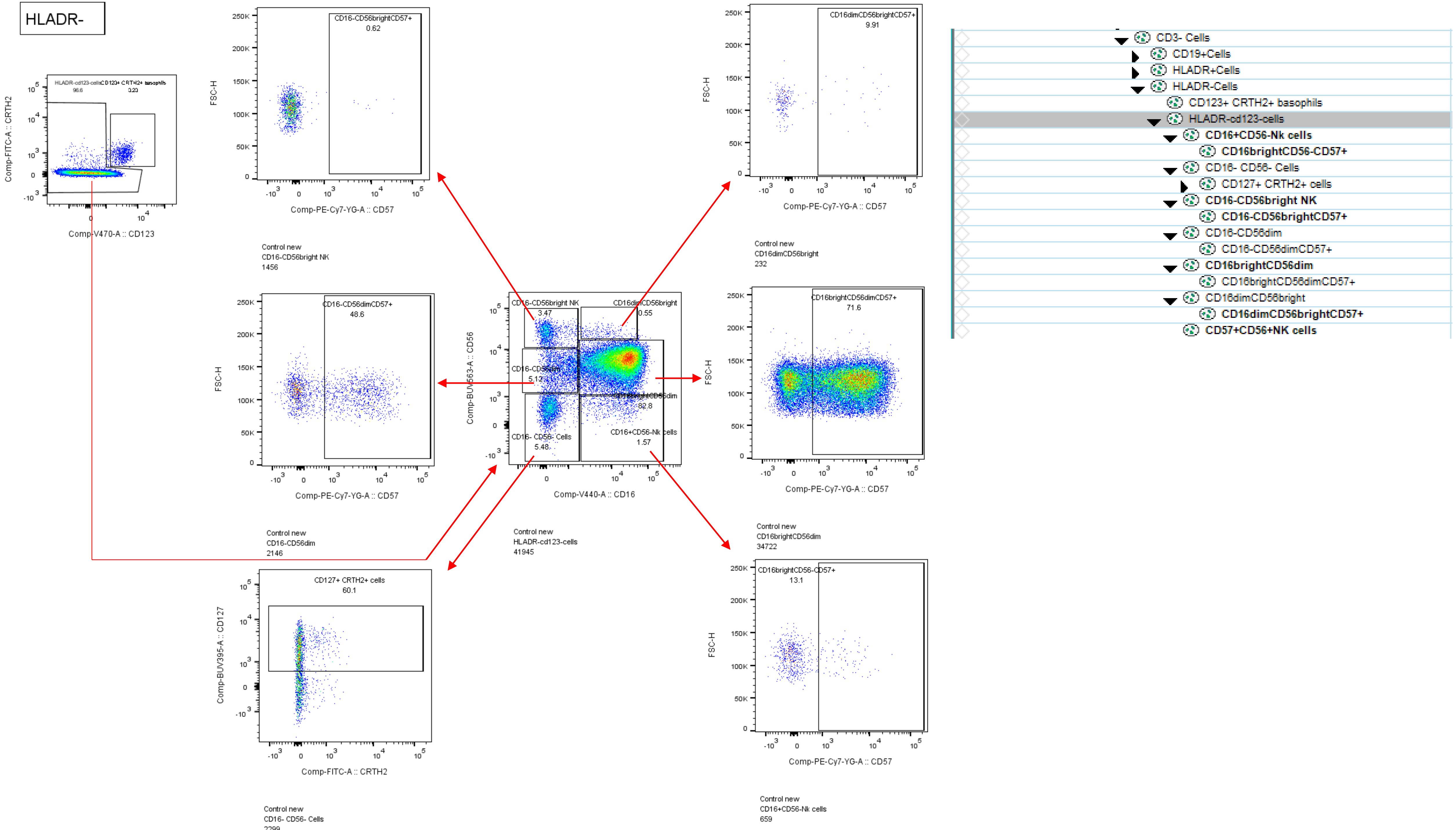

HLADR-
HLADR-cd123-cells 96.6
CD123+ CRTH2+ basophils 3.23
Comp-FITC-A :: CRTH2
Comp-V470-A :: CD123
CD16-CD56brightCD57+ 0.62
FSC-H
Comp-PE-Cy7-YG-A :: CD57
Control new
CD16-CD56bright NK
1456
CD16dimCD56brightCD57+ 9.91
Control new
CD16dimCD56bright
232
CD16-CD56dimCD57+ 48.6
Control new
CD16-CD56dim
2146
CD16-CD56bright NK 3.47
CD16dimCD56bright 0.55
CD16-CD56dim 5.12
CD16brightCD56dim 82.8
CD16- CD56- Cells 5.48
CD16+CD56-Nk cells 1.57
Comp-BUV563-A :: CD56
Comp-V440-A :: CD16
Control new
HLADR-cd123-cells
41945
CD16brightCD56dimCD57+ 71.6
Control new
CD16brightCD56dim
34722
CD127+ CRTH2+ cells 60.1
Comp-BUV395-A :: CD127
Comp-FITC-A :: CRTH2
Control new
CD16- CD56- Cells
CD16brightCD56-CD57+ 13.1
Control new
CD16+CD56-Nk cells
659
CD3- Cells
CD19+Cells
HLADR+Cells
HLADR-Cells
CD123+ CRTH2+ basophils
HLADR-cd123-cells
CD16+CD56-Nk cells
CD16brightCD56-CD57+
CD16- CD56- Cells
CD127+ CRTH2+ cells
CD16-CD56bright NK
CD16-CD56brightCD57+
CD16-CD56dim
CD16-CD56dimCD57+
CD16brightCD56dim
CD16brightCD56dimCD57+
CD16dimCD56bright
CD16dimCD56brightCD57+
CD57+CD56+NK cells

CD16-CD56-

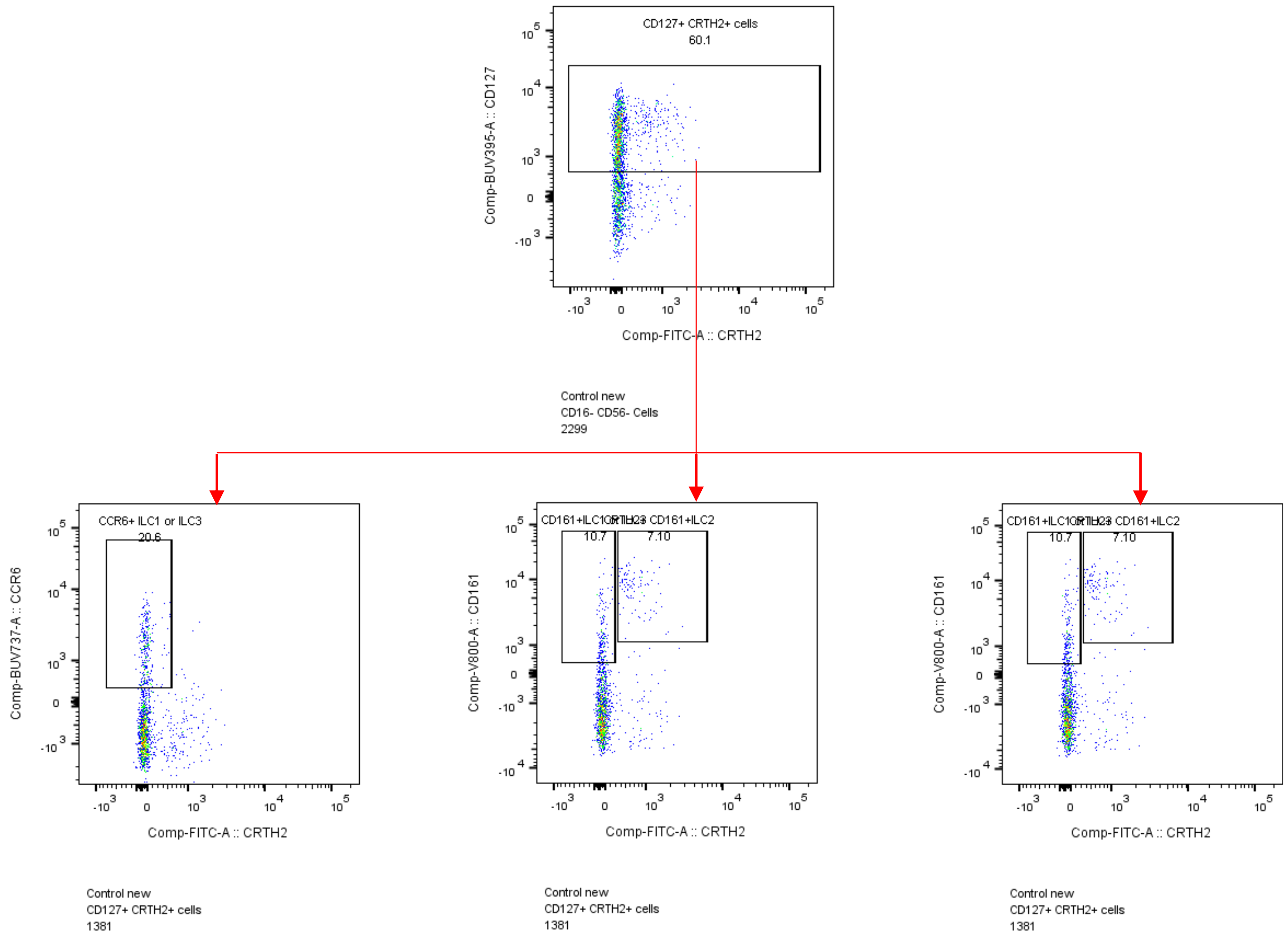

CD127+ CRTH2+ cells
60.1
Comp-BUV395-A :: CD127
Comp-FITC-A :: CRTH2
Control new
CD16- CD56- Cells
2299
CCR6+ ILC1 or ILC3
20.6
Comp-BUV737-A :: CCR6
Comp-FITC-A :: CRTH2
Control new
CD127+ CRTH2+ cells
1381
10.7
7.10
CRTH2+ CD161+ILC2
Comp-V800-A :: CD161
Comp-FITC-A :: CRTH2
Control new
CD127+ CRTH2+ cells
1381
10.7
7.10
CRTH2+ CD161+ILC2
Comp-V800-A :: CD161
Comp-FITC-A :: CRTH2
Control new
CD127+ CRTH2+ cells
1381

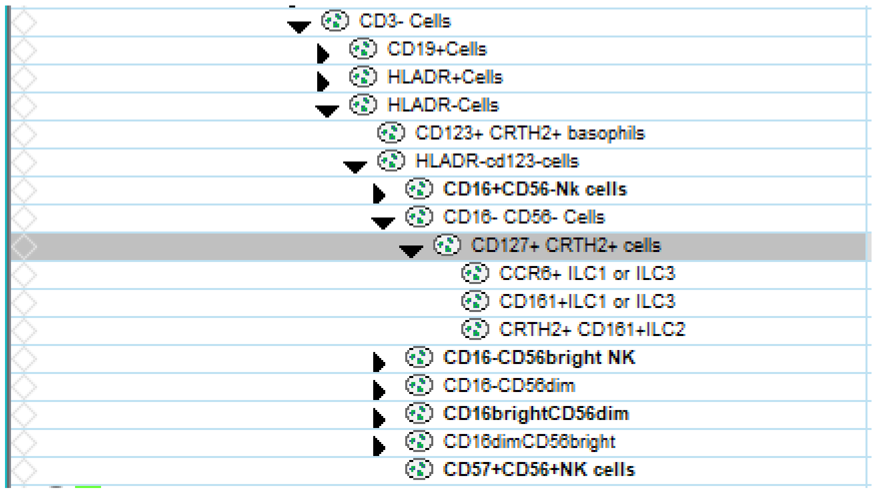

CD3- Cells
CD19+Cells
HLADR+Cells
HLADR-Cells
CD123+ CRTH2+ basophils
HLADR-cd123-cells
CD16+CD56-Nk cells
CD16- CD56- Cells
CD127+ CRTH2+ cells
CCR6+ ILC1 or ILC3
CD161+ILC1 or ILC3
CRTH2+ CD161+ILC2
CD16-CD56bright NK
CD16-CD56dim
CD16brightCD56dim
CD16dimCD56bright
CD57+CD56+NK cells
