# Supplementary material for: Novel approach to analysis of the immune system using an ungated model of immune surface marker abundance to predict health outcomes
Source: Immun Ageing. 2022 Aug 4;19:35. doi: 10.1186/s12979-022-00291-y (PMC9351261; doi:10.1186/s12979-022-00291-y)
Supplement: Supplementary file 3 — Additional file 3. Flow cytometry antibodies information. [file 12979_2022_291_MOESM3_ESM.docx]

| **Marker** | **Florochrome** | **Clone** | **Company** |
| --- | --- | --- | --- |
| CD34 | PE | 563 | BD |
| CD4 | BV750 | SK3 | BD |
| IgD | BV711 | IA6-2 | BD |
| CD8 | BV605 | SK1 | BD |
| CD14 | BV605 | M5E2 | BD |
| CD123 | BV480 | 9F5 | BD |
| CD19 | APC-R700 | HIB19 | BD |
| TCR γ/δ | APC-R700 | 11F2 | BD |
| CD27 | BV510 | L128 | BD |
| CD45 | BUV805 | HI30 | BD |
| CCR6 | BUV737 | 11A9 | BD |
| HLADR | BUV 661 | G46-6 | BD |
| CD56 | BUV563 | NCAM16.2 (NCAM 16) | BD |
| CD127 | BUV395 | HIL-7R-M21 | BD |
| CD25 | PE/Dazzle™ 594 | M-A251 | Biolegend |
| CD16 | BV421 | 3G8 | Biolegend |
| CRTH2 | FITC | BM16 | Biolegend |
| TCR Vδ2 | BV711 | B6 | Biolegend |
| CxCR3(CD183) | BV650 | G025H7 | Biolegend |
| CD3 | BV570 | UCHT1 | Biolegend |
| TCR Vα7.2 | APC-CY7 | 3C10 | Biolegend |
| CD38 | PE/Cy5 | HIT2 | Biolegend |
| CD45RO | PerCP/Cy5.5 | UCHL1 | Biolegend |
| CD161 | BV785 | HP-3G10 | Biolegend |
| CD57 | PE/Cy7 | HNK-1 | Biolegend |
| Zombie UV Fixable Viability Kit | BUV496 | - | Biolegend |
| TCR Vδ1 | APC-Vio770 | REA173 | Miltenyi Biotec |
| SLAN | APC | M-DC8 | Miltenyi Biotec |
